# Crystal structure and functional characterization of a cold-active acetyl xylan esterase (*Pb*AcE) from psychrophilic soil microbe *Paenibacillus* sp.

**DOI:** 10.1371/journal.pone.0206260

**Published:** 2018-10-31

**Authors:** Sun-Ha Park, Wanki Yoo, Chang Woo Lee, Chang Sook Jeong, Seung Chul Shin, Han-Woo Kim, Hyun Park, Kyeong Kyu Kim, T. Doohun Kim, Jun Hyuck Lee

**Affiliations:** 1 Unit of Polar Genomics, Korea Polar Research Institute, Incheon, Republic of Korea; 2 Department of Chemistry, College of Natural Science, Sookmyung Woman’s University, Seoul, Republic of Korea; 3 Department of Molecular Cell Biology, Samsung Biomedical Research Institute, Sungkyunkwan University School of Medicine, Suwon, Korea; 4 Department of Polar Sciences, University of Science and Technology, Incheon, Republic of Korea; Griffith University, AUSTRALIA

## Abstract

Cold-active acetyl xylan esterases allow for reduced bioreactor heating costs in bioenergy production. Here, we isolated and characterized a cold-active acetyl xylan esterase (*Pb*AcE) from the psychrophilic soil microbe *Paenibacillus* sp. R4. The enzyme hydrolyzes glucose penta-acetate and xylan acetate, reversibly producing acetyl xylan from xylan, and it shows higher activity at 4°C than at 25°C. We solved the crystal structure of *Pb*AcE at 2.1-Å resolution to investigate its active site and the reason for its low-temperature activity. Structural analysis showed that *Pb*AcE forms a hexamer with a central substrate binding tunnel, and the inter-subunit interactions are relatively weak compared with those of its mesophilic and thermophilic homologs. *Pb*AcE also has a shorter loop and different residue composition in the β4–α3 and β5–α4 regions near the substrate binding site. Flexible subunit movements and different active site loop conformations may enable the strong low-temperature activity and broad substrate specificity of *Pb*AcE. In addition, *Pb*AcE was found to have strong activity against antibiotic compound substrates, such as cefotaxime and 7-amino cephalosporanic acid (7-ACA). In conclusion, the *Pb*AcE structure and our biochemical results provide the first example of a cold-active acetyl xylan esterase and a starting template for structure-based protein engineering.

## Introduction

Xylan is the predominant hemicellulose found in the plant cell wall and the second most plentiful and renewable biopolymer after cellulose [1]. There has been growing interest in the enzymatic hydrolysis of xylan based on the potential for its hydrolyzed monomers to be converted into valuable products such as biofuels [2]. Because the structure of xylan contains a β-1,4-linked xylose backbone substituted with different side chains such as arabinosyl, glucuronosyl, feruloyl, *p*-coumaroyl, and acetyl residues [3–5], complete degradation of xylan requires the cooperation of several types of hemicellulolytic enzymes, including endo-xylanase, ß-xylosidase, α-arabinosidase, α-glucuronidase, and acetyl xylan esterase.

Among the various side chains, acetylation is the most common substitution of plant xylan. For example, approximately 70% of the xylose residues in hardwood xylan are acetylated at the C2 or C3 position [6]. Acetyl xylan esterases (EC 3.1.1.72; AXEs) catalyze the specific hydrolysis of the ester linkages between the xylose units and acetic acid, facilitating the access of main chain depolymerizing enzymes [7]. Based on their sequence similarities and structural folds, AXEs have been classified into nine carbohydrate esterase (CE) families, CE1–7, 12, and 16 in the Carbohydrate-Active Enzymes (CAZy) database (http://www.cazy.org/) [8]. The enzymes in these families display activities with various acetylated sugar substrates as well as with *p*-nitrophenyl acetate and *α*-naphtyl acetate [9–11]. In addition, AXEs belonging to the CE7 family also show deacetylation activity against cephalosporin antibiotics, which can be used to prepare important starting material for the production of semi-synthetic β-lactam antibiotics [9, 11–14].

Thus far, among the members of CE7, seven enzymes have been biochemically characterized, five of which have had their crystal structure solved. These are derived from *Bacillus pumilus*; *Bacillus subtilis*; the thermophiles *Thermoanaerobacterium* sp. JW/SL YS485 and *Thermotoga maritima*; and a soil metagenome (PDB id: 2XLB, 1ODS, 3FCY, 3M81, and 6FKX, respectively) [9, 14–16]. These enzymes employ the canonical catalytic triad Ser-His-Asp and share typical esterase α/β hydrolase folds, but they exhibit several distinct structural features compared to those of the other CE family members, including their high oligomeric state. These enzymes are mainly donut-shaped hexamers consisting of a trimer of dimers. Six active sites are directed toward the center of the hexameric ring structure, which displays a narrow entrance tunnel and serves to confer substrate selectivity by limiting the access of large substrates. Other conserved structural features include an N-terminal extension containing two α-helices and one β-strand; a three-helix insertion after strand β6; and a loop insertion before β4, called the β-interface loop. Previous studies of the *T*. *maritima* CE7 (*Tm*AcE) have shown that these features are all involved in inter-subunit interfacing and are essential for thermal stability, oligomerization, and catalytic activity [17, 18].

The psychrophilic strain *Paenibacillus* sp. R4 was previously isolated from active-layer soil in Council, Alaska. The genome of this strain was analyzed, and several putative esterase/lipase/thioesterase family genes were annotated by similarity searches of sequence databases. We identified a novel acetyl xylan esterase (*Pb*AcE) belonging to the CE7 family from this strain. Despite the considerable potential for industrial applications of cold-active enzymes that retain high catalytic activity at low temperatures, no AXEs in the CE7 family derived from psychrophilic microorganisms have yet been studied. Rather, all characterized AXEs in the family exhibit moderate or high temperature optima (30–90°C) [9, 12, 19]. In this study, we determined the three-dimensional structure of *Pb*AcE and investigated its biochemical properties, including its substrate specificity toward several acetylated compounds and cephalosporin antibiotics.

## Material and methods

### Cloning of *Pb*AcE

The *Pb*AcE gene was amplified by PCR from the genomic DNA of *Paenibacillus* sp. R4 using the forward primer 5′- CTGCCATATGCCTAATGTAGATATGCCTTT-3′ and the reverse primer 5′- CTGGCTCGAGTTACAGATAAGCTTCTATGA-3′. The DNA fragment was ligated into expression vector pET-28a (Novagen, Madison, WI, USA) via *Nde*I and *Xho*I restriction sites. The expression construct, which introduced a cleavable hexa-His N-terminal tag, was used to transform *Escherichia coli* BL21(DE3) for expression.

#### Protein expression and purification

Cells were grown in LB medium supplemented with kanamycin at 50 μg ml^−1^ at 37°C to an optical density of 0.5 at 600 nm, at which point *Pb*AcE expression was induced by the addition of 0.5 mM isopropyl-1-thio-β-d-galactopyranoside (IPTG). Protein expression continued overnight at 25°C before collection by centrifugation. Cell pellets were resuspended in buffer A (50 mM sodium phosphate, pH 8.0, 300 mM NaCl, 5 mM imidazole, and 0.2 mg ml^−1^ lysozyme) and lysed by ultrasonic treatment, followed by centrifugation at 16,000 rpm for 1 h at 4°C. The resulting lysate was purified by Ni^2+^ affinity chromatography (Qiagen, Hilden, Germany) and elution with an imidazole gradient (20–300 mM). The collected fraction was concentrated using Amicon ultracentrifuge filters (Ultracel-3K; Millipore, Darmstadt, Germany), digested with thrombin, and further separated on a Superdex 200 column (GE Healthcare, Piscataway, NJ, USA) pre-equilibrated with buffer B (50 mM Tris-HCl, pH 8.0, and 150 mM NaCl). Peak fractions containing *Pb*AcE were collected and resolved using 12% SDS-PAGE.

### Crystallization and data collection

Purified protein was concentrated to 101.6 mg ml^-1^. The mosquito high-throughput crystallization robot (TTP Labtech, UK) was used to identify initial crystallization conditions. *Pb*AcE was screened at 293 K in 96-well sitting drop plates (Emerald Bio, Bainbridge Island, WA, USA) using commercially available kits, such as the MCSG I-IV (Microlytic, Burlington, VT, USA), SG-1 (Molecular Dimension, USA), Wizard Classic I-IV (Emerald Bio), and SaltRx and Index (Hampton Research, Aliso Viejo, CA, USA). A 200-nl drop of protein solution was mixed with an equal volume of reservoir solution and equilibrated against 80 μl of reservoir solution. Crystals of *Pb*AcE were grown within 1–2 days at 293 K under conditions of 1.8 M sodium phosphate monobasic monohydrate, potassium phosphate dibasic, pH 5.0 (SaltRx #E8). The optimal crystal was harvested and soaked in *N*-paratone oil (Hampton Research) for cryo-protection. The crystal was flash-cooled and then mounted under a liquid nitrogen stream. X-ray diffraction data for *Pb*AcE were collected at 2.1-Å resolution on a beamline BL5-C from Pohang Accelerator Laboratory (PAL; Pohang, Korea). The data set containing 200 images was integrated and scaled using *HKL-2000* [20]. Detailed crystal parameters and data collection statistics are summarized in Table 1.

**Table 1:** X-ray diffraction data collection and refinement statistics.

Data set	*Pb*AcE
X-ray source	PAL-5C beam line
Detector	ADSC Quantum 315r
Wavelength (Å)	0.9796
Space group	*C*2
Cell dimensions (Å,°)	a = 153.8, b = 141.7, c = 105.4, α = γ = 90, β = 104.5
Resolution (Å)	50.0–2.10 (2.14–2.10)
Wilson B-factor (Å^2^)	23.5
Total reflections	445004
Unique reflections	121844 (6275)
Average I/σ (I)	27.2 (5.7)
R_merge_^a^	0.118 (0.549)
Redundancy	3.7 (4.0)
Completeness (%)	96.8 (99.9)
**Refinement**	
Resolution range (Å)	49.4–2.10 (2.16–2.10)
No. of reflections of working set	115533 (8000)
No. of reflections of test set	6247 (439)
No. of amino acid residues	1896
No. of water molecules	767
Average B-factor (Å^2^) (protein)	36.4
Average B-factor (Å^2^) (solvent)	68.8
rmsd B-factor for bonded atom (Å)	2.897
rmsd bond length (Å)	0.017
rmsd bond angle (°)	1.806
Ramachandran outlier (%)	0.00
Ramachandran favoured (%)	96.2
Rotamer outlier (%)	3.18
C-beta outlier	13
Clashscore	1.05
Overall score	1.44
*R*_cryst_^b^	0.198 (0.366)
*R*_free_^c^	0.254 (0.386)

^a^ *R*_merge_ = ∑ | <I>–I | /∑<I>.

^b^ *R*_cryst_ = ∑ | |Fo|—|Fc| | /∑|Fo|.

^c^ *R*_free_ calculated with 5% of all reflections excluded from refinement stages using high-resolution data.

Values in parentheses refer to the highest resolution shells.

### Structure determination and refinement

The structure of *Pb*AcE was solved by molecular replacement using the *MOLREP* program from the *CCP4* suite. To identify molecular replacement search models, a PSI-BLAST search was performed using the PDB database. The results showed that five *Tm*AcE structures (PDB codes: 3M81, 5GMA, 1VLQ, 3M83, and 5HFN) were listed as the top five solutions. Among these, we selected the *Tm*AcE structure with the highest resolution coordinates (PDB code 5FDF; 1.76 Å resolution) to solve the *Pb*AcE structure by molecular replacement. The cross-rotation search with this template model returned clear hits. The hit of the rotation function with the highest score was used for the translation function. The model gave a strong single peak in the translation function, and the solution was used for further refinement and model building [18, 21, 22]. The correct sequence was manually fitted using *Coot* and refined with *REFMAC5* and *PHENIX* [23–25]. After iterative rebuilding and refinement, the final structure had an *R*_cryst_ value of 19.8% and *R*_free_ value of 25.4%. Model quality was analyzed using *MolProbity* [26]. Structure determination and refinement statistics are given in Table 1. Structural representations were generated using *PyMOL* [27]. The coordinate and structure factors of *Pb*AcE were deposited in the RCSB Protein Data Bank under accession id 6AGQ (S1 File).

### AUC analysis

Sedimentation velocity analysis of *Pb*AcE was performed at 20°C with an XL-A analytical ultracentrifuge (Beckman Coulter, Brea, CA, USA). The protein solution (0.5 mg/ml) was dissolved in a buffer of 20 mM Tris-HCl, pH 8.0, and 150 mM NaCl. The sample and reference sectors of the dual-sector epon centerpiece were filled with the *Pb*AcE protein solution and the buffer, respectively, and the cell was centrifuged at a rotor speed of 45,000 rpm. The sedimentation profile was monitored over time at 280 nm, and the experimental data were analyzed using the SEDFIT program [28, 29].

### Functional characterization of *Pb*AcE

Assays of the activity and thermal stability of *PbAcE* were performed at or above room temperature to compare the results with those of previously characterized esterases [9, 12, 13]. For substrate specificity analysis of *Pb*AcE, *p*-nitrophenyl (*p*NP) ester derivatives with different acyl chain lengths [*p*-nitrophenyl acetate (*p*NP-C_2_), *p*-nitrophenyl butyrate (*p*NP-C_4_), *p*-nitrophenyl hexanoate (*p*NP-C_6_), *p*-nitrophenyl octanoate (*p*NP-C_8_), *p*-nitrophenyl decanoate (*p*NP-C_10_), and *p*-nitrophenyl dodecanoate (*p*NP-C_12_)] were used as substrates. Reactions containing 10 μg of *Pb*AcE and 250 μM of substrate were incubated for 5 min at room temperature. The amount of *p*-nitrophenol released from the hydrolysis of *p*NP ester derivatives by *Pb*AcE was quantified by measuring absorbance at 405 nm. For *α*-*β*-naphthyl ester derivatives, reactions containing 10 μg of *Pb*AcE and 50 μM of substrates were incubated for 5 min at room temperature. The hydrolase activity was monitored by measuring absorbance at 315 nm. The hydrolase activity of *Pb*AcE toward various substrates was assessed using colorimetric analysis with phenol red as a pH indicator. Substrates included carbohydrate esters [10 mM of α-d-glucose penta-acetate, 10 mM of *N*-acetyl-d-glucosamine, 2% (w/v) of cellulose acetate and acetyl xylan], tertiary alcohol esters (100 mM of tert-butyl acetate, linalyl acetate, and α-terpinyl acetate), lipids [1% (v/v) of glyceryl tri-butyrate/-oleate, olive oil, and fish oil], and antibiotic-related compounds (100 mM of cefotaxime, 7-ACA, and cephalosporin C). Each reaction mixture containing the above substrates was incubated with 100 μg of *Pb*AcE at 37°C for the indicated time. The temperature-specific active properties of *Pb*AcE were studied by incubating 10 μg of *Pb*AcE at temperatures ranging from 4 to 37°C. After 1 h of incubation, *p*NP-C_2_ was added to the incubated mixture at a final concentration of 250 μM. The thermal stability and chemical stability of *Pb*AcE were investigated by incubating 10 μg of *Pb*AcE at various temperatures (37, 50, 60, and 70°C) with various chemical compounds (10% or 30% ethanol, 30% propanol, 1% Tween 20, 1% Triton X-100, 1% SDS, and 10 mM PMSF). After 1 h of incubation, *p*NP-C_2_ was added at final concentration of 250 μM. The kinetic parameters of *Pb*AcE were investigated using *p*NP-C_2_ and *p*NP-C_4_ as substrates with 5 μg of *Pb*AcE. The absorbance at 405 nm was monitored for 10 min, and the initial linear measurements were used for determining the slope of the initial velocity. The molar extinction coefficient for *p*-nitrophenol was 16,400 M^-1^ cm^-1^ at pH 8.0. The Michaelis–Menten constant [30], maximum velocity (*V*_*max*_), turnover rate (*k*_*cat*_), and catalytic efficiency (*k*_*cat*_/*K*_*M*_) were calculated from double reciprocal plots (GraphPad Prism 6.0 software). For comparing the thermal stabilities of wild-type *Pb*AcE and the L144R mutant, 1 mg/ml of each enzyme was incubated at 70°C for 1 h. Heated samples of 10 μl were collected every 20 min, and the residual activity was measured using *p*NP-C_2_ as a substrate. All reactions mentioned above were carried out in 20 mM Tris-HCl and 150 mM NaCl, pH 7.4. Absorbances were measured using an Epoch 2 microplate reader (BioTek, USA).

### Preparation of acetyl xylan

Xylan (10 g) from beechwood (Sigma-Aldrich, St. Louis, MO, USA) was dissolved in 250 ml of DMSO at 55°C for 24 h. Next, 0.4 g of potassium borate was added to the xylan solution while stirring at 55°C, followed by 200 ml acetic anhydride, which was added slowly over 5 min. After 4 h of incubation at 55°C, the mixture was dialyzed against tap water at 4°C for 5 days, followed by dialysis against distilled water for 1 day.

### Immobilization of *Pb*AcE

For cross-linked enzyme aggregate (CLEA) preparation, 500 μg *Pb*AcE was precipitated with 80% ammonium sulfate and cross-linked by 25 mM glutaraldehyde, followed by gentle agitation for 12 h. After centrifugation, the pellet was resuspended, washed repeatedly, and finally stored in 20 mM Tris-HCl and 150 mM NaCl, pH 7.4, for further analysis. For reusability assay, *Pb*AcE CLEAs were repeatedly used in a new enzyme reaction after extensive washing until no activity was detected from the supernatant. For mCLEA preparation, different amounts of *Pb*AcE were precipitated and cross-linked using the method described above but in the presence of 500 μg of magnetic nanoparticles (MNPs). PbAcE mCLEAs were recovered from the reaction solution by magnet and washed by gentle agitation in 20 mM Tris-HCl and 150 mM NaCl, pH 7.4. For assessment of the hydrolase activity of *Pb*AcE CLEAs and *Pb*AcE mCLEAs, *p*NP-C_2_ was used as a substrate.

### Acetylation activity assay

To synthesize acetyl xylan, 2% (w/v) xylan from beechwood (Sigma-Aldrich) and *Pb*AcE CLEAs prepared from 500 μg *Pb*AcE were dissolved in hexane and 1 M acetic acid to a final volume of 1 ml. After incubation at 37°C with continuous shaking, 1 μl of synthesized acetyl xylan was directly analyzed by gas chromatography (Agilent 7890A, Agilent Technologies, Santa Clara, CA, USA). The gas chromatographer was installed with a HP-5 capillary column (20 m × 0.18 mm i.d., 0.18 μm film thickness, Agilent). The injector and detector temperatures were 190°C. Samples (1 μl) were injected with a 1:20 split ratio. The initial oven temperature was set to 35°C (1 min) and then programmed to increase to 160°C at a 10°C/min ramping rate.

## Results and Discussion

### Characterization of *Pb*AcE

The recombinant *Pb*AcE protein was expressed and purified to apparent homogeneity as described in the Materials and methods. The purified *Pb*AcE showed a homogenous band with a molecular mass of ~36 kDa following SDS-PAGE (S1A Fig). Analytical ultracentrifugation (AUC) was performed to determine the oligomeric state of *Pb*AcE. The resulting sedimentation coefficient distribution confirmed that, like other members of the CE7 family, *Pb*AcE exists as a hexamer with a corresponding molecular mass of 234 kDa. (S1B Fig). In the phylogenetic analysis, *Pb*AcE was clustered with CE7 family esterases (S2 Fig).

### Structure of *Pb*AcE

**Fig 1:**
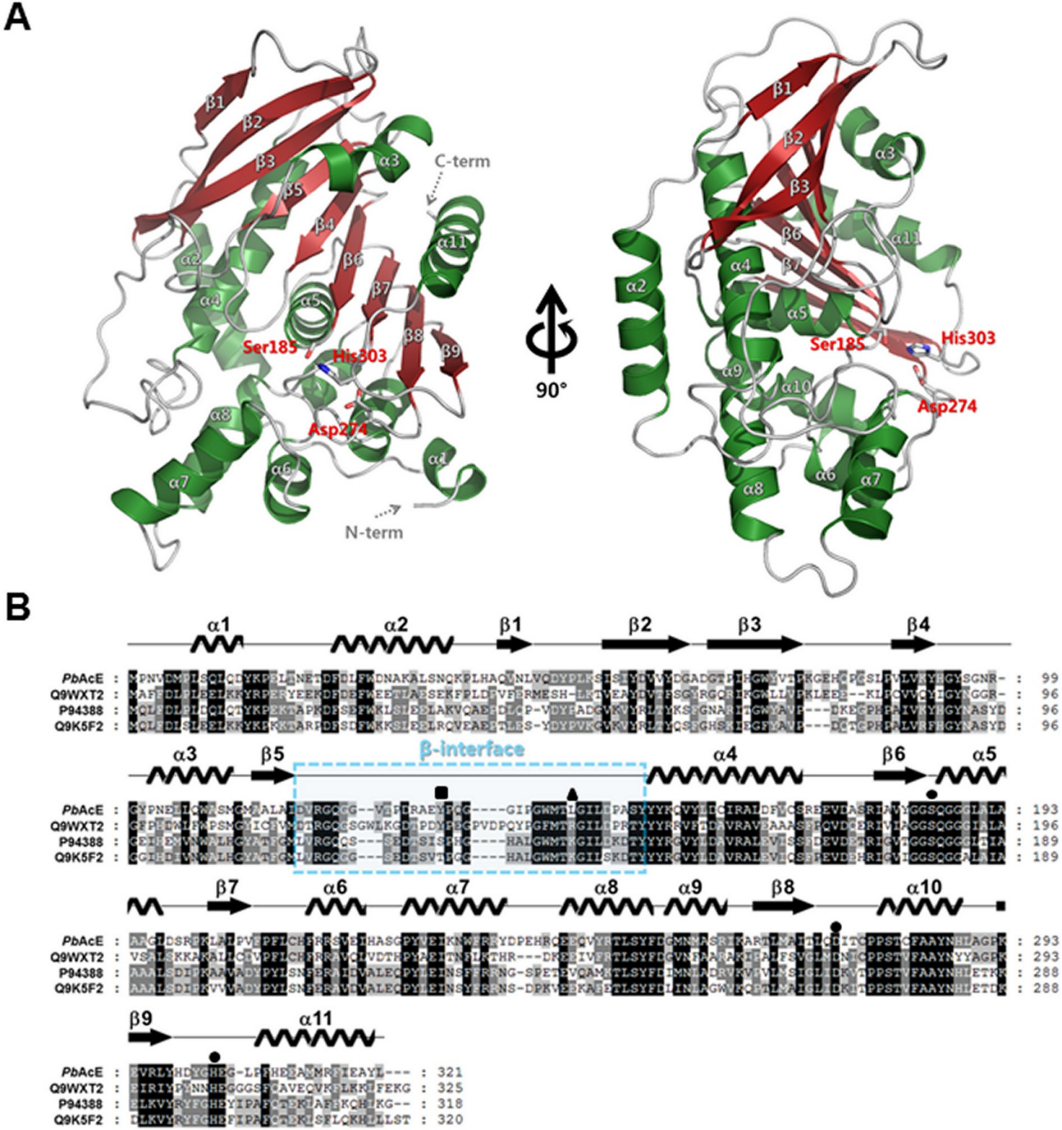
Crystal structure of *Pb*AcE and multiple sequence alignment. (A) Overall structure of *Pb*AcE is shown in front and 90° rotated views. Ribbon representation of *Pb*AcE, with the β-strands in forest green and α-helices in red. The conserved catalytic triad residues are shown as grey stick models. (B) Sequence alignment of *Pb*AcE with secondary structure. Aligned sequences include *Pb*AcE, *Tm*AcE (UniProtKB id: Q9WXT2), *Bs*AcE (UniProtKB id: P94388), and *Bp*AcE (UniProtKB id: Q9K5F2). The β5–α4 loop region (residues 119–152), called the β-interface, is boxed in sky blue. The Tyr133 and Leu144 residues located in β-interface region are indicated above the alignment residues with a black rectangle and triangle, respectively. The catalytic triad residues of Ser185, Asp274, and His303 are indicated with black circles.

To obtain structural insight into the substrate binding of *Pb*AcE, we first sought to solve the *Pb*AcE crystal structure. After initial crystallization screening, the monoclinic-shaped crystals of *Pb*AcE grew under the conditions of 1.8 M sodium phosphate monobasic monohydrate, potassium phosphate dibasic, pH 5.0, within 1–2 days (S1C Fig). The best crystal with a size of 0.3 mm was diffracted to ~ 2.1-Å resolution (S1D Fig). The structure of *Pb*AcE was solved by the molecular replacement method using an acetyl transferase from *T*. *maritima* (PDB id: 5FDF; sequence identity: 44%) as a search model [18]. The crystal structure of *Pb*AcE belongs to the *C*2 space group and contains six molecules in the asymmetric unit, forming a donut-shaped hexamer. The central tunnel of the donut-shaped hexamer has a diameter of about 18.5 Å. Each monomer of *Pb*AcE is composed of 11 α-helices and 9 β-strands. In detail, the central large β-sheet consists of an antiparallel β-sheet (β1–β3) and a parallel β-sheet (β4–β6), and α-helices surround the central β-sheet. Moreover, *Pb*AcE also has an N-terminus extended by α1–α2 helices. These features of the α/β hydrolase fold are common for other CE7 members (Fig 1). A Dali structural homology search with *Pb*AcE showed that cephalosporin C deacetylase from *B*. *subtilis* (PDB id: 1L7A; sequence identity: 43%) and acetyl xylan esterase from *T*. *maritima* (PDB id: 3M81; sequence identity: 46%) returned the top Z scores of 46.7 (S1 Table) [9, 31].

### Active site of *Pb*AcE

**Fig 2:**
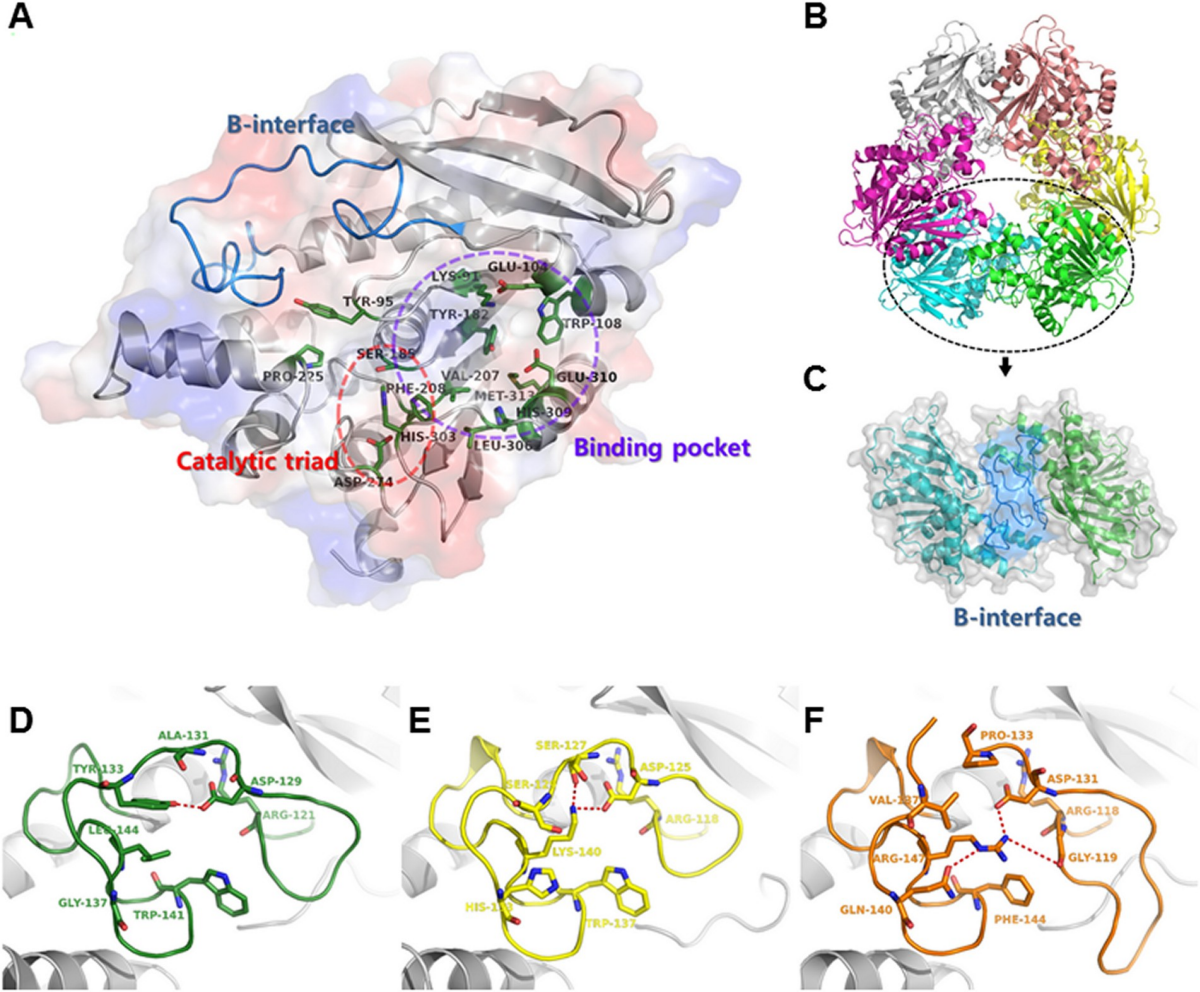
Active site and β-interface of *Pb*AcE. (A) Active site and substrate binding site are circled in salmon and purple, respectively. Side chains of catalytic triad and residues located at substrate binding site are indicated by stick models (forest green). The β-interface region is represented in marine. (B) *Pb*AcE forms a donut-shaped hexamer containing a trimer of dimers. (C) The dimer interface between each pair of monomers contains the β-interface region (marine). Close-up view of β-interfaces of *Pb*AcE (D), *Bs*AcE (E), and *Tm*AcE (F) depicted in forest green, yellow, and orange, respectively. Specific residues that affect the conformation of the β-interface are shown as stick models. Hydrogen bonds in the β-interface are represented as red dashed lines.

The conserved catalytic triad residues of Ser185, Asp274, and His303 are located inside the hexamer tunnel, with a neighboring putative substrate binding site. This hydrophobic putative substrate binding site is composed of β4, β6, and β7 strands and α3 and α11 helices (Fig 2A). It consists of several specific residues, including Lys91, Glu104, Trp108, Tyr182, Val207, Phe208, Leu306, His309, Glu310, and Met313. Unlike the core residues of the catalytic triad, the specific residues that comprise the substrate binding site vary by species (S3 Fig). In comparison with the acetate-bound *Bs*AcE (PDB id: 1ODS) and substrate analog 2-(2-oxo-1,3-dihydroindol-3-yl)acetate (OIA)-bound *Tm*AcE (PDB id: 5JIB), several different residues were identified [14, 32]. The residue Trp108, located on the edge of the substrate binding site, was conserved in both structures. However, the nearby residues of Phe208, Val207, and Tyr182 show obvious differences across proteins. The bound acetate and OIA molecules are spatially limited by the α7–α8 loop and the β4–α3 loop. The conserved residues of Tyr95 and Pro225 are located on each loop, respectively. These distinct differences in substrate binding sites may affect substrate specificity. Moreover, there is another factor to consider in terms of substrate specificity: like other CE7 family proteins, in the hexameric state, the N-terminus of each *Pb*AcE molecule is located at the entrance of the tunnel. For this structural reason, the N-terminus acts as gatekeeper for various substrates.

### β-interface of *Pb*AcE

In the hexamerization of CE7 family proteins, there is a key interface between each pair of monomers called the β-interface [14, 17]. This region, located on the β5–α4 loop, forms an antiparallel β-strand-like interaction. In *Tm*AcE, which has high thermal stability, deletion of the β-interface results in a significant reduction in thermal stability [18, 33]. The β-interface has also been identified at the dimer interfaces of *Pb*AcE (Fig 2B and 2C). This interface contains several conserved interactions. However, *Pb*AcE shows several differences in this region. Specifically, *Bs*AcE (PDB id: 1ODT) and *Tm*AcE (PDB id: 5JIB) have lysine and arginine residues in the β5–α4 loop region, respectively [14, 32]. The lysine or arginine residue stretches to the corresponding residue on the other side of the subunit and interacts to hold the dimer together. However, in *Pb*AcE, this residue is substituted for leucine (Fig 2D, 2E and 2F). The leucine residue is shorter than lysine and arginine residues, preventing dimer residues from interacting with each other and forming hydrogen bonds. Moreover, Gly137 of *Pb*AcE is substituted for the histidine and glutamine residues in *Bs*AcE and *Tm*AcE, respectively. These different interactions in the β5–α4 loop region may affect the stability and flexibility of the hexamer. *Pb*AcE is derived from a psychrophilic microbe, and it is known that psychrophilic enzymes are more flexible [34, 35]. Thus, analysis of salt bridges was carried out using the ESBRI server with a cutoff distance of 5 Å [36]. The hexamer complex and monomers of *Tm*AcE (PDV id: 3M81) contain 354 and 58 ionic side-chain interactions, respectively, whereas the hexamer complex and monomers of *Pb*AcE contain only 256 and 38, respectively. This indicates that the structure of *Tm*AcE contains many more salt bridges than that of *Pb*AcE. These numerous interactions in *Tm*AcE make it highly thermostable, while the reduced interactions in *Pb*AcE make it more flexible, which may allow it to function at low temperatures.

### Role of gatekeeper in *Pb*AcE

**Fig 3:**
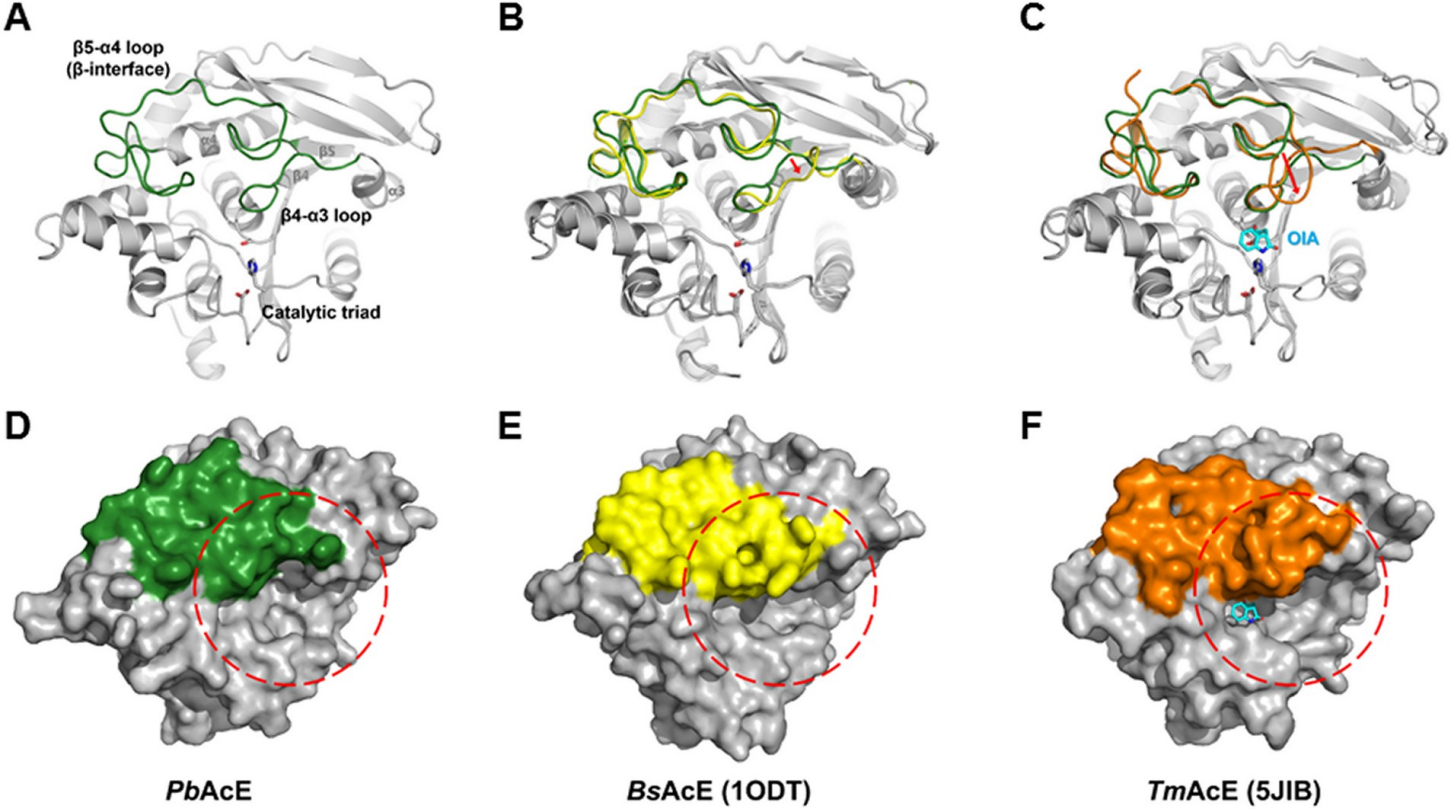
Comparison of different entrance conformations. (A) The β4–α3 and β5–α4 loop (β-interface) regions form a substrate gate in *Pb*AcE (forest green). (B) Superposition with *Bs*AcE (yellow) shows the difference in the β4–α3 loop region. (C) Superposition with *Tm*AcE (orange) shows the difference in the β5–α4 loop (β-interface) region. A bound OIA molecule is shown as a cyan stick model. Surfaces of *Pb*AcE (D), *Bs*AcE (E), and *Tm*AcE (F) represent the entrances for substrates, circled with red dashed lines. Only the β4–α3 and the β5–α4 loop regions are colored as above the figure, while the remaining protein is in gray.

Structural comparisons of *Pb*AcE with other AcEs showed significant differences in the size of the cleft area located near the catalytic triad (Fig 3). *Pb*AcE has a relatively more open and larger cleft in comparison with those of *Bs*AcE and *Tm*AcE. Structural alignment with *Bs*AcE showed that, unlike in *Pb*AcE, the β4–α3 loop region tended to cover the cleft, with the residue Tyr95, located on the β4–α3 loop of *Bs*AcE, extending to the cleft site. By contrast, in the case of *Tm*AcE, the β5–α4 loop protrudes and covers the cleft. The Trp124 located on the β5–α4 loop stretches to the cleft and constrains the space. It is thought that the rearrangement of these residues is related to the movements necessary for the interactions involved in ligand binding. The B-factor distribution analysis of the *Pb*AcE structure showed that the values of the β4–α3 (residues 93–101) and β5–α4 (residues 119–152) loop regions were lower than the average for the whole *Pb*AcE structure (S4 Fig). In contrast, in the case of *Tm*AcE and *Bs*AcE, those regions have much higher values than the corresponding averages (values of 24.12 and 11.22 Å^2^, respectively). Notably, the *Pb*AcE β4–α3 and β5–α4 loop structures are one and six residues shorter than those of *Tm*AcE, respectively. These shortened gatekeeper loops may expand the substrate binding site area and create a more open active site conformation. Collectively, the results of this structural analysis suggest that the β4–α3 and β5–α4 loop regions could act as gatekeepers in AcEs. In particular, the relatively large cleft of *Pb*AcE should facilitate substrate binding at low temperatures. In addition, it offers a spatial advantage in accommodating a wider variety of substrates.

### Substrate specificity of *Pb*AcE

**Fig 4:**
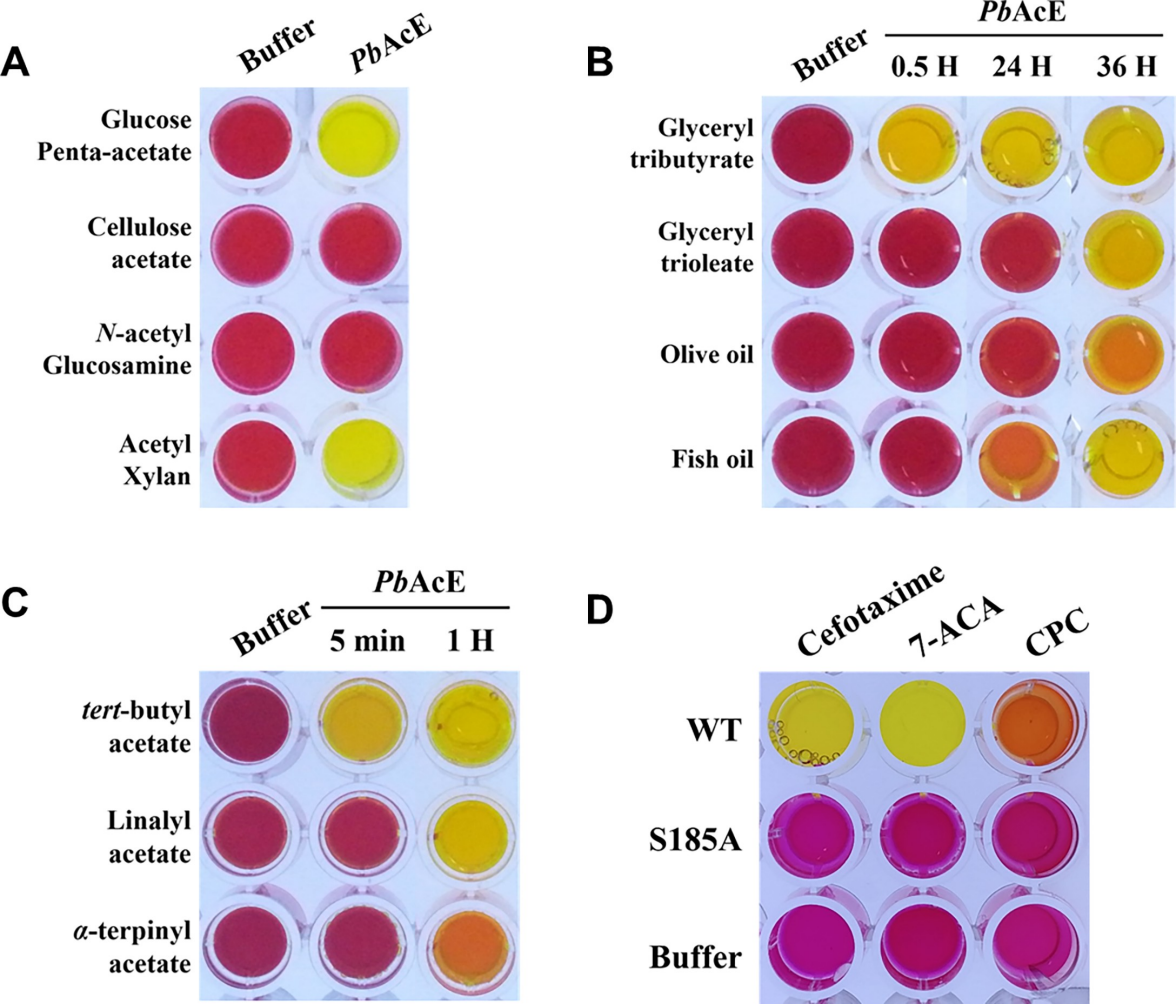
Substrate specificity of *Pb*AcE. (A) A pH shift assay was performed to measure the hydrolytic activity of acetylated carbohydrate substrates. The hydrolytic activities toward (B) lipids and (C) tertiary alcohol esters were also examined under the indicated reaction times. (D) The hydrolysis of antibiotic-related compounds by *Pb*AcE wild-type and S185A inactive mutant: 7-ACA, 7-aminocephalosporanic acid; CPC, cephalosporin C. Acetic acid released in the enzyme reaction changed the solution color from red to yellow.

*Pb*AcE was examined for its ability to remove acetyl groups from four acetylated carbohydrate substrates: glucose penta-acetate, cellulose acetate, *N*-acetyl glycosamine, and acetyl xylan. A colorimetric assay based on a pH indicator, phenol red, was performed. In this assay system, color changes to yellow are induced when the pH decreases due to the release of acetic acid from the substrate. As shown in Fig 4A, *Pb*AcE was active on glucose penta-acetate, one of the simplest acetylated carbohydrates, which is consistent with other previously reported acetyl esterases [9, 11, 12]. In addition, *Pb*AcE showed activity toward acetyl xylan, clearly demonstrating that *Pb*AcE is indeed an acetyl xylan esterase, not an acetyl esterase. However, in contrast to acetyl xylan esterases belonging to families CE1, CE4, and CE5, *Pb*AcE did not show activity toward cellulose acetate, indicating that *Pb*AcE likely does not belong to those families and has different substrate specificity [37]. When *N*-acetyl glycosamine was used as a substrate, no color change was observed, probably because *Pb*AcE selectively hydrolyzes ester bonds but not amide bonds.

Next, the activity of *Pb*AcE towards glyceryl esters (glyceryl tributyrate and glyceryl trioleate), oils (olive oil and fish oil), and tertiary alcohol esters (tertiary-butyl acetate, linalyl acetate, and α-terpinyl acetate) was investigated. The enzyme hydrolyzed all tested substrates, with particularly efficient hydrolysis of glyceryl tributyrate and tertiary-butyl acetate (Fig 4B and 4C). In addition, *Pb*AcE notably exhibited significant deacetylation activity against β-lactam-related substrates, such as cefotaxime, 7-amino cephalosporanic acid (7-ACA), and cephalosphorin C (Fig 4D). An S185A catalytic triad mutant was completely inactive toward all tested substrates. As reported for other CE7 members, *Pb*AcE showed higher activity for 7-ACA than for cephalosphorin C [9, 11, 12, 14]. The deacetylation activity, as well as the low-temperature activity of *Pb*AcE, could reduce the thermal degradation of cephalosporins, allowing this enzyme to be efficiently used for the semi-synthesis of new antibiotics.

**Fig 5:**
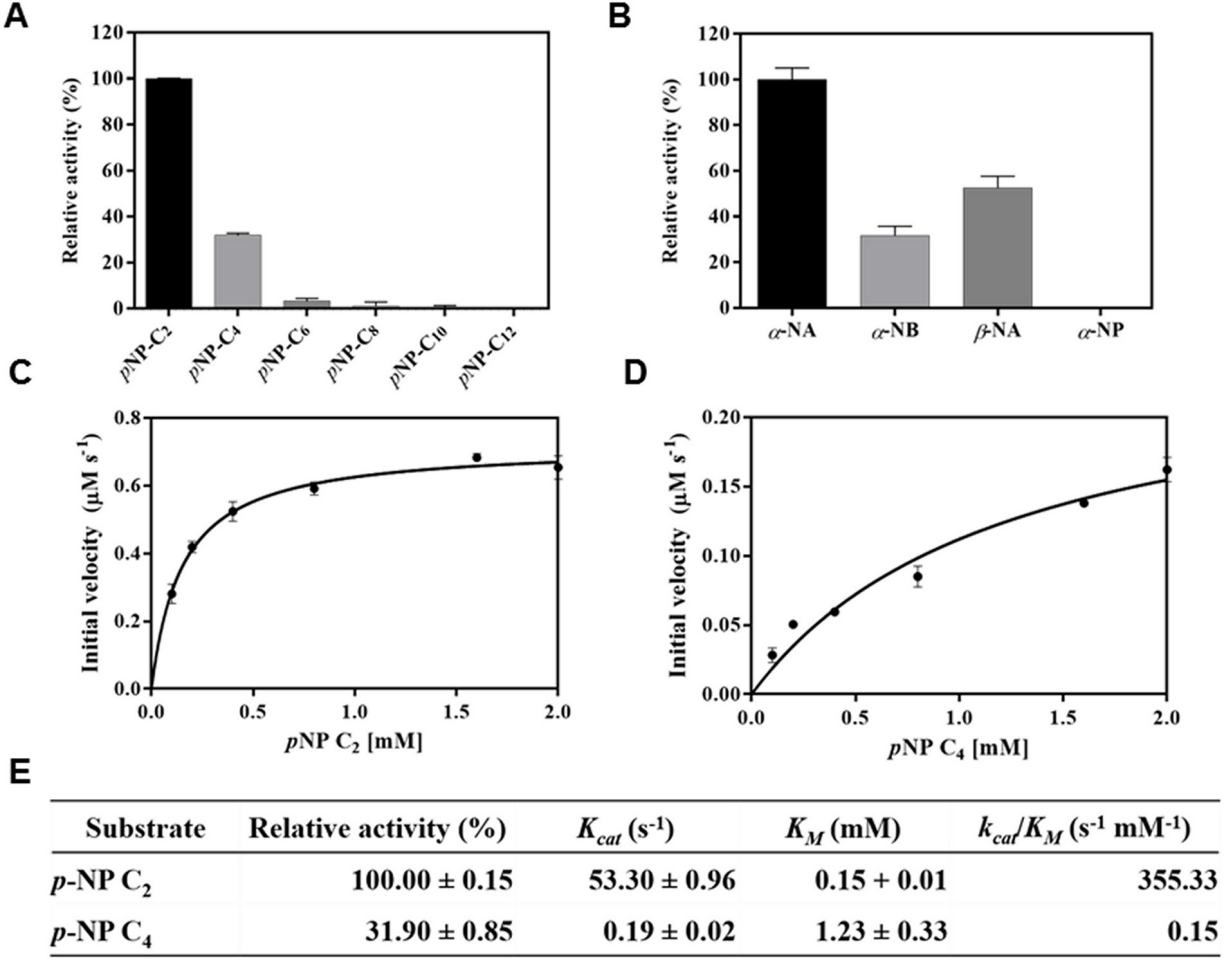
Hydrolytic activities toward *p*NP and naphthyl esters. The relative enzyme activity of *Pb*AcE for *p*-nitrophenyl (*p*NP) esters with varying acyl chain lengths from C2 to C8 (A) and *α*-,*β*-naphthyl ester derivatives (B). The change in the initial rate of the reaction at different concentrations of (C) *p*NP-acetate and (D) *p*NP-butyrate are shown. (E) Relative activities and kinetic parameters of *Pb*AcE towards these two substrates were determined from the initial rate measurements. The highest activity obtained was set as 100%. All measurements were performed in triplicate.

To obtain more information regarding the substrate specificity of *Pb*AcE, enzyme activities were investigated using *p*-nitrophenyl (*p*NP) esters with varying acyl chain lengths, from C2 to C8 (Fig 5A). *Pb*AcE strongly prefers *p*NP-acetate (C2), followed by *p*NP-butyrate (C4), while no or little activity was detected against *p*NP-esters with acyl chain lengths longer than C6. Similar observations have been reported in previous studies on other members of the CE7 family [9, 12]. Next, we investigated the substrate preference of *Pb*AcE on naphthyl derivatives (Fig 5B). The highest activity was detected against α-naphthyl acetate, followed by β-naphthyl acetate and α-naphthyl butyrate, but activity was not observed against α-naphthyl phosphate. Additionally, initial kinetic studies were performed for *p*NP-C2 and *p*NP-C4 (Fig 5C and 5D). The values of the kinetic parameters are shown in Fig 5E; *Pb*AcE exhibited a *K*_m_ approximately 1.5-fold lower and a catalytic efficiency (*k*_cat_/*K*_m_) more than 2000 times higher for *p*NP-C2 compared with those of *p*NP-C4.

### Effects of temperature and organic solvents on *Pb*AcE activity

**Fig 6:**
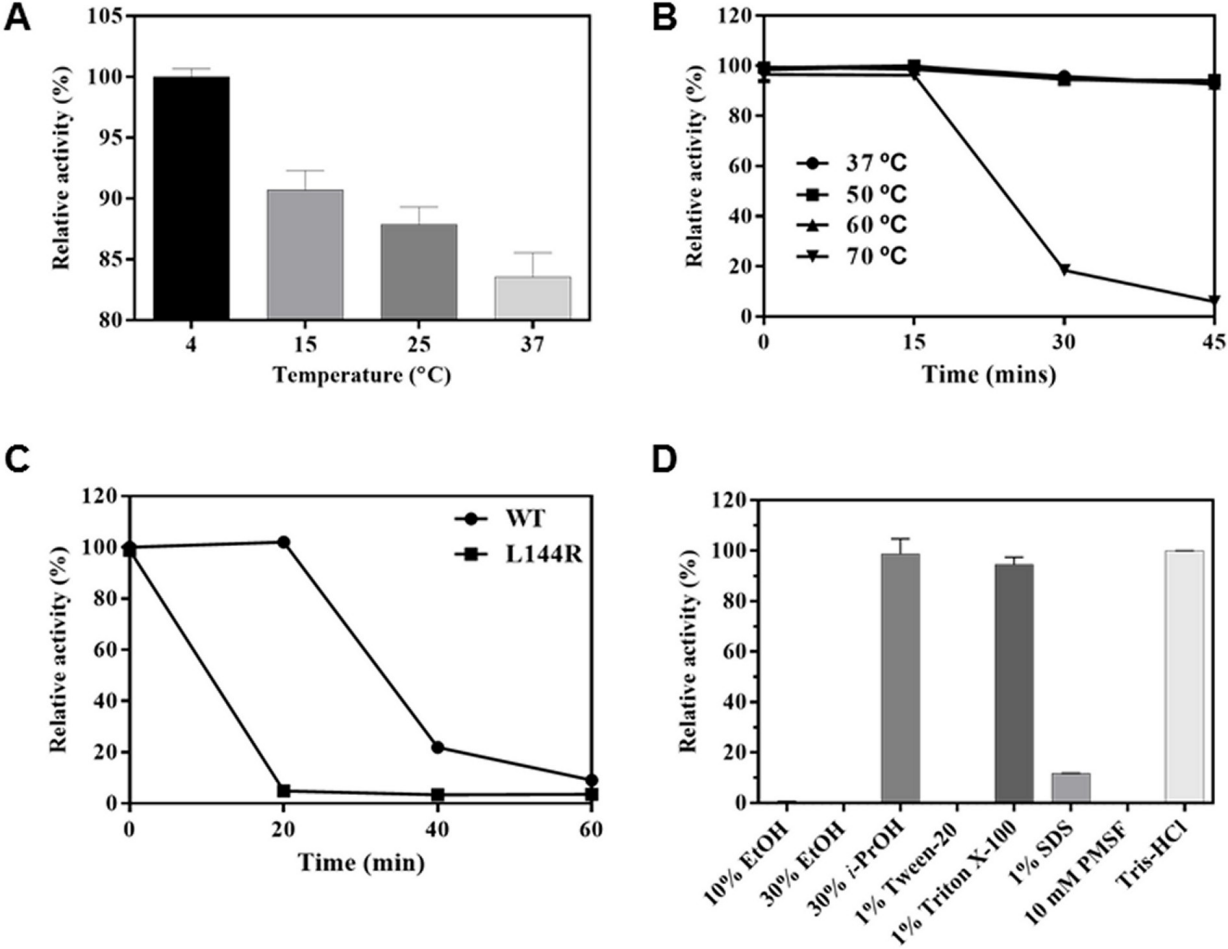
Effects of temperature and organic solvents on the activity of *Pb*AcE. (A) Enzyme activity was measured at various temperatures. (B) Thermal stability was determined by assaying residual enzyme activity after incubation of *Pb*AcE for different time periods at the temperatures indicated. (C) After incubation of *Pb*AcE wild-type and L144R mutant at 70°C, residual activities were measured. (D) Chemical stability of *Pb*AcE was investigated after exposure to various organic solvents for 1 h and determination of residual activities, expressed relative to the original activity. All measurements were performed in triplicate using *p*NP-C_2_ as a substrate.

The effect of temperature on *Pb*AcE activity was investigated using *p*NP-acetate as a substrate (Fig 6A). Some CE7 members with high sequence similarity to *Pb*AcE are moderately to highly thermostable (temperature optima: 30–90°C) [9, 12, 19]. However, *Pb*AcE, which is derived from a psychrophilic microorganism, showed the highest activity at 4°C, suggesting that its structural and biochemical properties are optimized to low temperatures. The thermal stability of *Pb*AcE was investigated by measuring the residual activity after incubation of the enzyme for different time intervals at temperatures ranging from 37 to 70°C (Fig 6B). The enzyme was fully stable at temperatures below 60°C, but, after 30 min of incubation at 70°C, over 80% of the activity disappeared. From our sequence alignment and structural analysis, we found that leucine residue 144, located on the β-interface, is substituted for the arginine in *Tm*AcE, a thermostable acetyl xylan esterase [18]. It was therefore of interest to generate a *Pb*AcE L144R mutant and investigate the effect of the mutation on thermal stability. When we compared the thermal stability of wild-type and L144R mutant *Pb*AcE, the wild-type protein was more stable than the L144R mutant (Fig 6C). After 20 min of incubation at 70°C, the activity of wild-type *Pb*AcE was almost unchanged, while the L144R mutant completely lost its activity. In the *Pb*AcE structure, the L144 residue forms a hydrophobic interaction with Y133, stabilizing the β-interface loop structure. This result suggests that the β-interface loop structure is highly associated with the stability or activity of *Pb*AcE.

The effect of organic solvents on the activity of *Pb*AcE was also investigated, with the enzyme retaining over 95% of its original activity in the presence of 30% isopropanol and 1% Triton X-100, a nonionic detergent (Fig 6D). Taken together, its psychrophilic activity and high organic solvent stability suggest that *Pb*AcE could be a suitable candidate for industrial biocatalysis.

### Immobilization of *Pb*AcE

**Fig 7:**
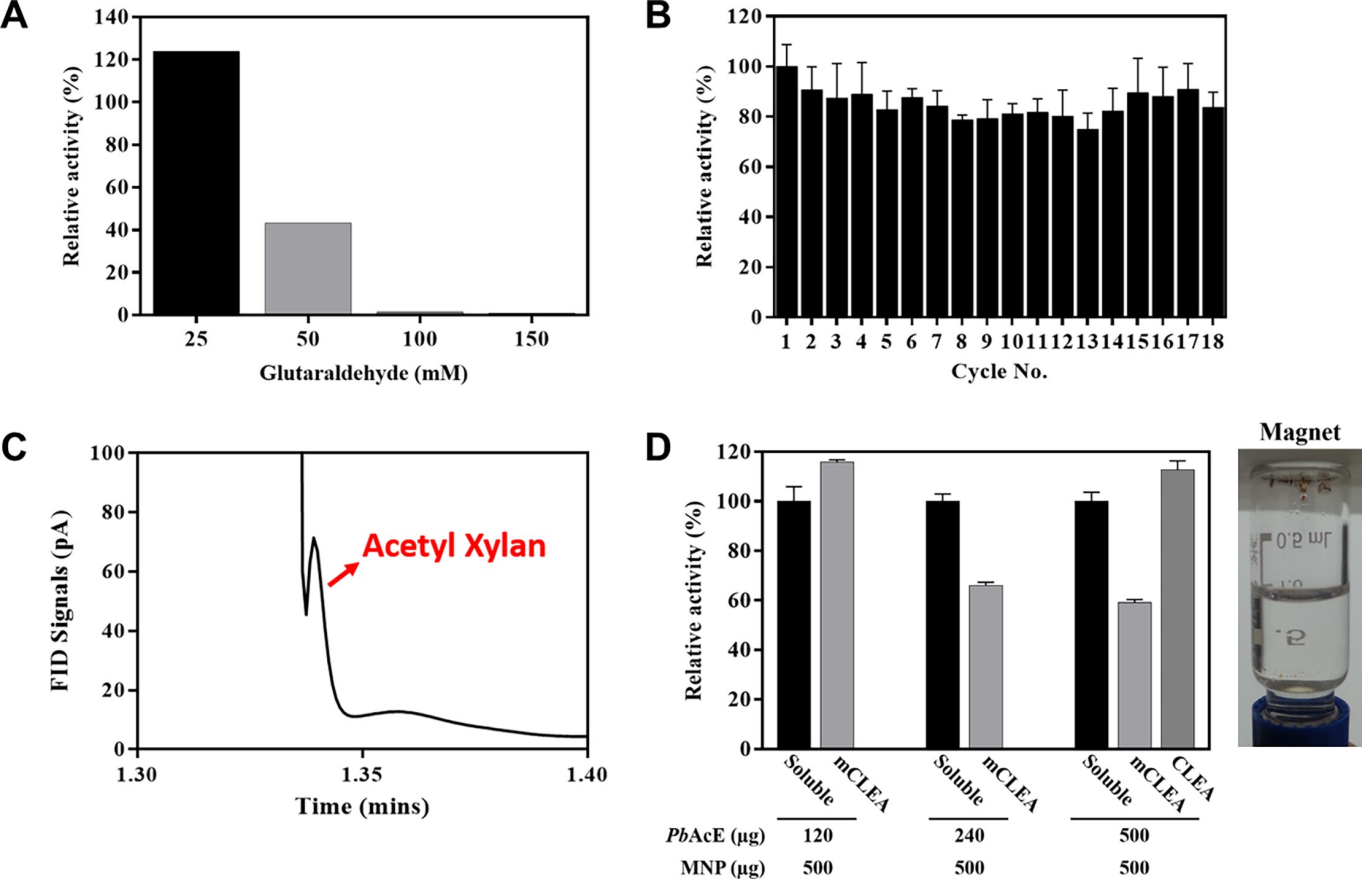
Immobilization of *Pb*AcE. (A) The relative activities of *Pb*AcE CLEAs cross-linked by different concentrations of glutaraldehyde. (B) Reusability of *Pb*AcE CLEAs was compared to that of the soluble enzyme for 18 cycles. (C) Acetylation activity of *Pb*AcE CLEAs on xylan was observed via gas chromatography. (D) The relative activities of *Pb*AcE CLEAs and *Pb*AcE mCLEAs were compared to that of the soluble enzyme. The *Pb*AcE mCLEAs were prepared with different amounts of *Pb*AcE and 500 μg of MNPs. The activity of soluble *Pb*AcE was set as 100%. All measurements were performed in triplicate using *p*NP-C_2_ as a substrate.

Efficient recyclability and increased stability are critical factors for the cost-effective use of enzymes in industrial processes. In order to improve its potential for industrial applications, *Pb*AcE was immobilized as CLEAs by solvent precipitation and cross-linking with glutaraldehyde [38–40]. The first step was to find the optimum concentration of glutaraldehyde for preparation of CLEAs with enhanced activity. When we tested different concentrations of glutaraldehyde, the *Pb*AcE CLEAs cross-linked by 25 mM glutaraldehyde showed the highest activity (Fig 7A). Moreover, the *Pb*AcE CLEAs showed good activity recovery and reusability after 18 cycles of washing and retained more than 75% of their initial activity (Fig 7B). Interestingly, based on gas chromatography analysis, *Pb*AcE CLEAs were found to also have acetylation activity as well as deacetylation activity (Figs 4A and 7C). These findings suggest that it will be possible to produce industrially valuable acetyl xylan through homogeneous acetylation based on the substrate specificity of the enzyme. Additionally, *Pb*AcE CLEAs were immobilized on MNPs using different concentrations of protein and MNPs. MNPs have attracted considerable attention as a support for enzyme immobilization, as they facilitate the easy separation of CLEAs from reaction products without time-consuming centrifugation steps upon application of an external magnetic field [41, 42]. The activity of the magnetic CLEAs (mCLEAs) was compared to those of free *Pb*AcE and *Pb*AcE CLEAs. As a result, we determined the optimal conditions for the preparation of mCLEAs (120 μg *Pb*AcE and 500 μg MNPs) with higher activity than those of free *Pb*AcE and *Pb*AcE CLEAs (Fig 7D).

## Conclusions

Here, we present the first crystal structure of a cold-adapted acetyl xylan esterase from the psychrophilic soil microbe *Paenibacillus* sp. R4. The determination of structural information, together with biochemical studies, provided a detailed understanding of the mechanism of this enzyme’s cold-temperature activity and broad substrate specificity. These results further provide novel insights into protein-engineering strategies for the development of particularly useful enzymes for effectively removing acetyl groups in the pharmaceutical and biofuel industries.

## Supporting information

S1 Fig Recombinant *Pb*AcE protein purification, crystallization, and X-ray diffraction data collection. (PDF)

S2 Fig Phylogenetic analysis of *Pb*AcE. (PDF)

S3 Fig Structural comparisons of active sites between *Pb*AcE and its homologs. (PDF)

S4 Fig B-factor analysis of AcEs. (PDF)

S1 Table Selected structural homologs of *Pb*AcE from a DALI search (DALI-Lite server). (PDF)

S1 File Validation report for PDB code 6AGQ. (PDF)
