# Supplementary material for: Crystal structure and functional characterization of a cold-active acetyl xylan esterase (PbAcE) from psychrophilic soil microbe Paenibacillus sp
Source: PLoS One. 2018 Oct 31;13(10):e0206260. doi: 10.1371/journal.pone.0206260 (PMC6209228; doi:10.1371/journal.pone.0206260)
Supplement: S1 Fig — (PDF) [file pone.0206260.s001.pdf]

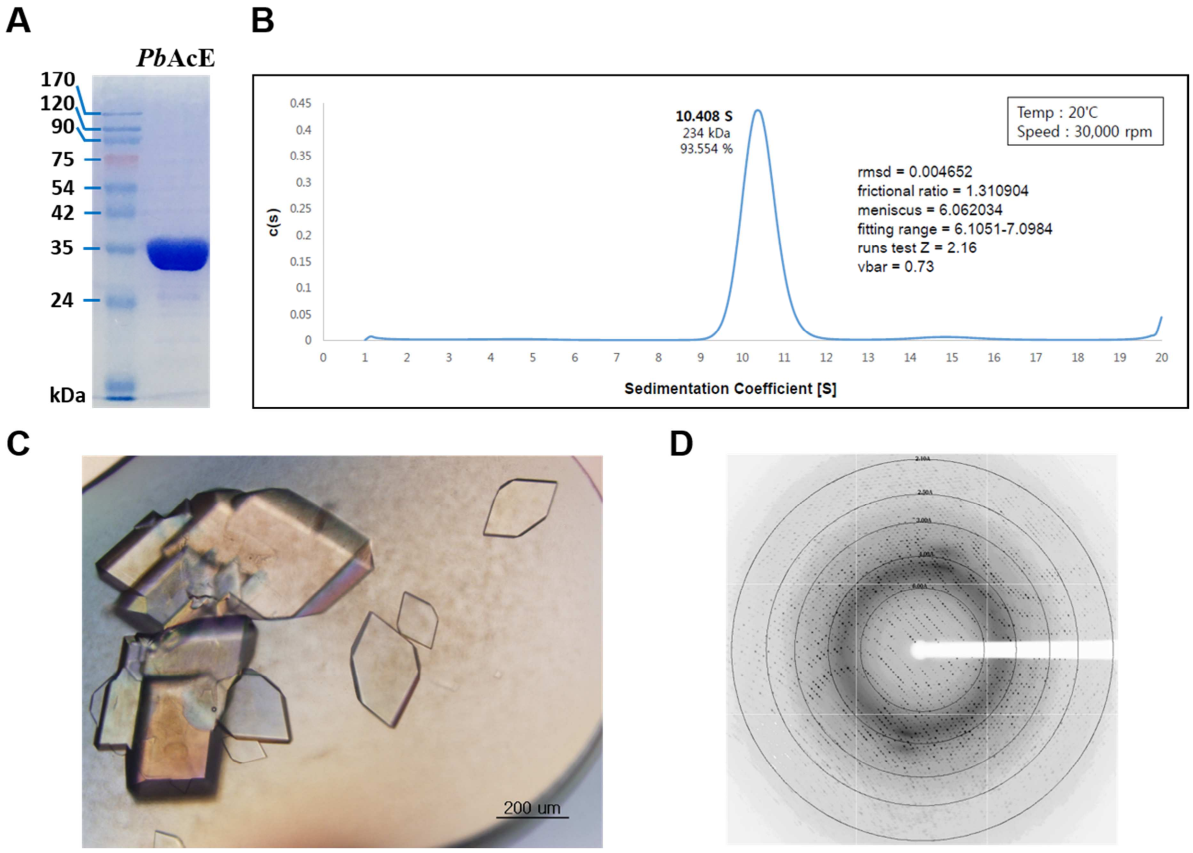

S1 Fig. Recombinant *Pb*AcE protein purification, crystallization, and X-ray diffraction data collection. (A) Purified *Pb*AcE protein (10 µg) was loaded and visualized using 12% SDS-PAGE (B) Analytical ultracentrifugation (AUC) experiments using 0.5 mg/ml *Pb*AcE gave a mass of 234 kDa (sedimentation coefficient of 10.408 S and frictional ratio of 1.31), indicating that *Pb*AcE is a stable hexamer in solution. (C) Crystals of *Pb*AcE used for X-ray diffraction data collection. (D) A representative X-ray diffraction pattern of the *Pb*AcE crystal is shown with a maximum resolution limit of 2.1 Å.
