# Supplementary material for: Crystal structure and functional characterization of a cold-active acetyl xylan esterase (PbAcE) from psychrophilic soil microbe Paenibacillus sp
Source: PLoS One. 2018 Oct 31;13(10):e0206260. doi: 10.1371/journal.pone.0206260 (PMC6209228; doi:10.1371/journal.pone.0206260)
Supplement: S2 Fig — (PDF) [file pone.0206260.s002.pdf]

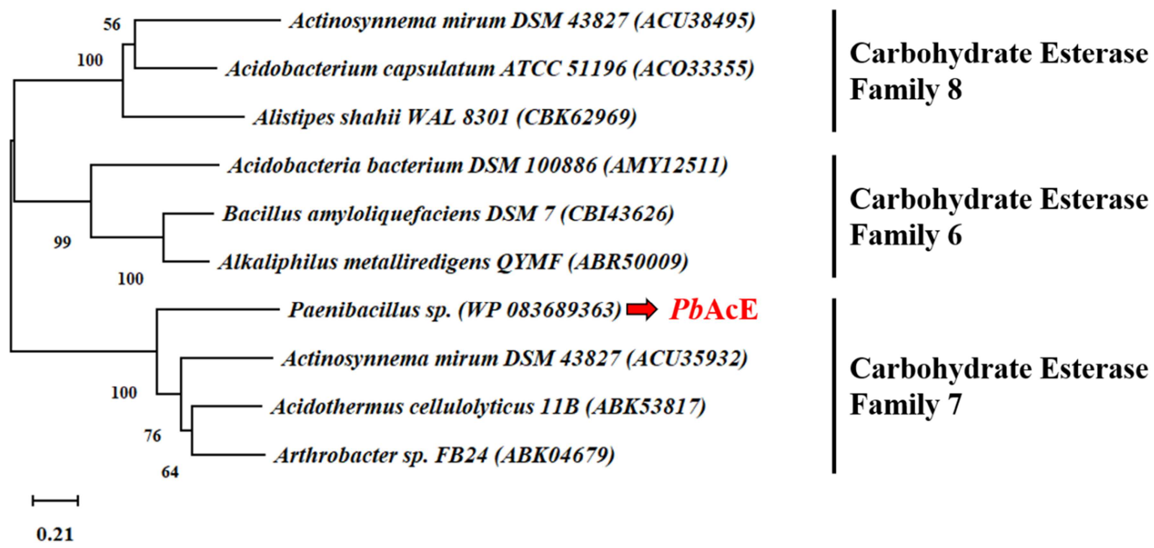

S2 Fig. Phylogenetic analysis of *Pb*AcE. Carbohydrate esterase family 6–8 protein sequences were chosen for comparison, and the phylogenetic tree was constructed using MEGA software 7.0 with the neighbor-joining method.
