# Supplementary material for: Crystal structure and functional characterization of a cold-active acetyl xylan esterase (PbAcE) from psychrophilic soil microbe Paenibacillus sp
Source: PLoS One. 2018 Oct 31;13(10):e0206260. doi: 10.1371/journal.pone.0206260 (PMC6209228; doi:10.1371/journal.pone.0206260)
Supplement: S3 Fig — (PDF) [file pone.0206260.s003.pdf]

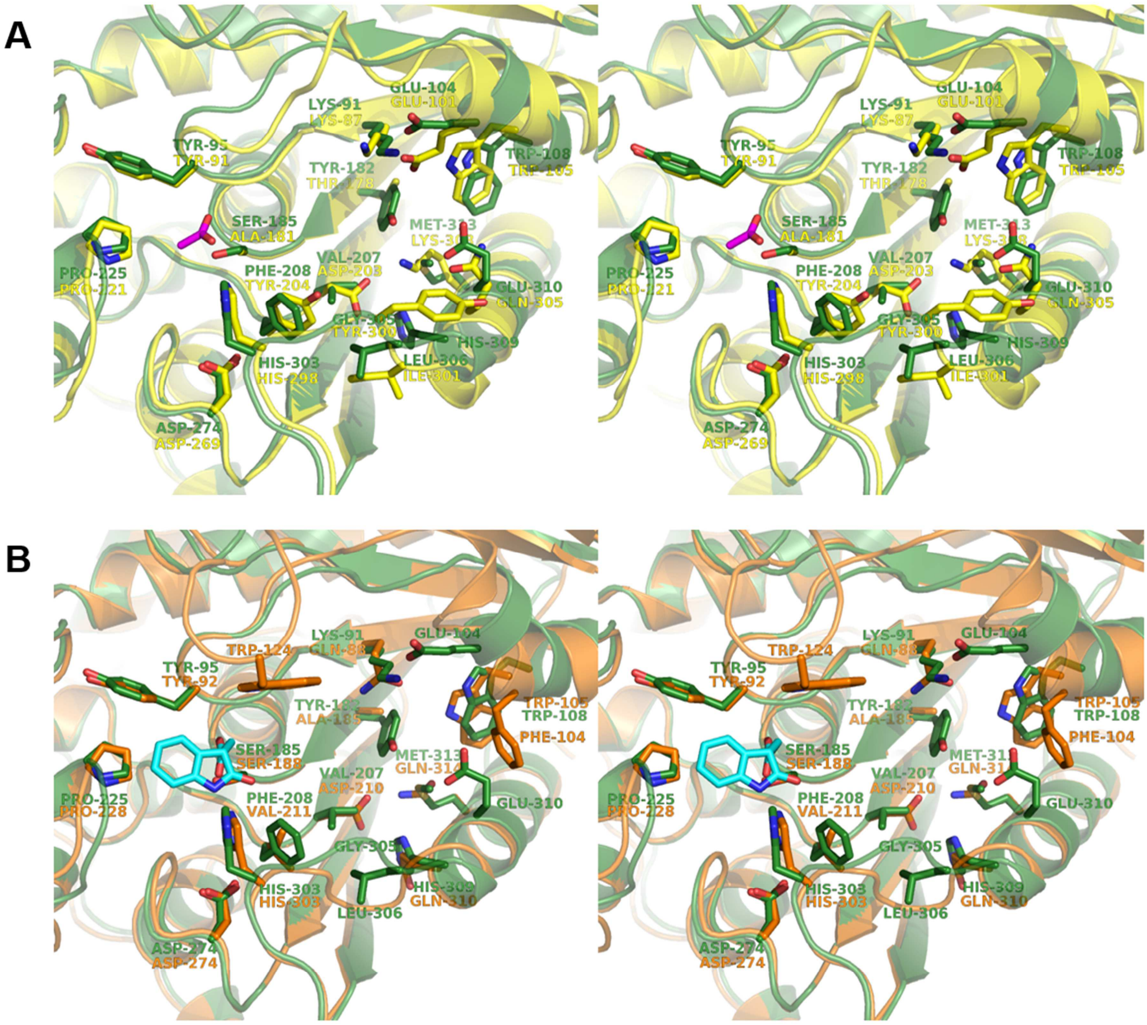

S3 Fig. Structural comparisons of active sites between *Pb*AcE and its homologs. (A) Structural superposition between *Pb*AcE (forest green) and acetate (magenta)-bound *Bs*AcE (PDB id: 1ODT; yellow). (B) OIA (cyan)-bound *Tm*AcE (orange) is superposed with *Pb*AcE. All molecules are represented as cartoon and stick models in stereo-view.
