# Supplementary material for: Crystal structure and functional characterization of a cold-active acetyl xylan esterase (PbAcE) from psychrophilic soil microbe Paenibacillus sp
Source: PLoS One. 2018 Oct 31;13(10):e0206260. doi: 10.1371/journal.pone.0206260 (PMC6209228; doi:10.1371/journal.pone.0206260)
Supplement: S4 Fig — (PDF) [file pone.0206260.s004.pdf]

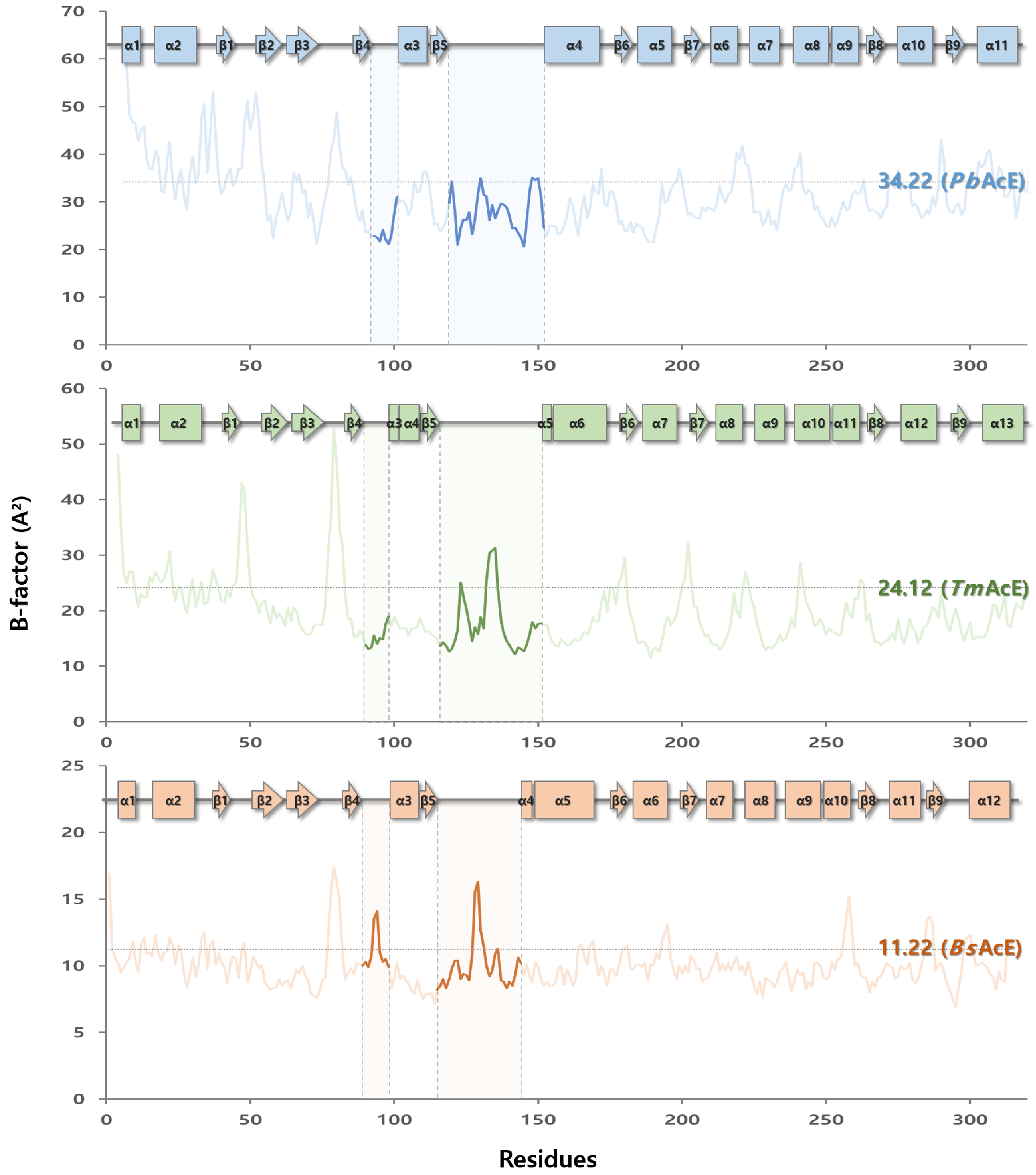

S4 Fig. B-factor analysis of AcEs. Plots of crystallographic B-factor values against residue numbers for *Pb*AcE (PDB code: 6AGQ; sky blue), *Tm*AcE (PDB code: 5JIB; green) and *Bs*AcE (PDB code: 1ODT; orange). The β4–α3 and β5–α4 loop regions of *Pb*AcE are boxed in sky blue, and the corresponding regions of *Tm*AcE and *Bs*AcE are boxed in light green and light orange, respectively. Each average B-factor is represented by dashed lines.
