# Supplementary material for: Crystal structure and functional characterization of a cold-active acetyl xylan esterase (PbAcE) from psychrophilic soil microbe Paenibacillus sp
Source: PLoS One. 2018 Oct 31;13(10):e0206260. doi: 10.1371/journal.pone.0206260 (PMC6209228; doi:10.1371/journal.pone.0206260)
Supplement: S1 Table — (PDF) [file pone.0206260.s005.pdf]

S1 Table. Selected structural homologs of *Pb*AcE from a DALI search (DALI-Lite server).

Protein	PDB id	DALI score	R.m.s.d. (Å)	Sequence % identity with *Pb*AcE (aligned residue number/total residue number)	Reference
Cephalosporin C deacetylase from *Bacillus subtilis*	1L7A	46.5	1.2	44% (308/318)	Not yet published
Acetyl esterase from *Thermotoga maritima*	3M81	46.5	1.5	46% (309/322)	(2012) Proteins 80: 1545-1559
Acetyl xylan esterase from *Bacillus pumilus*	2XLB	46.2	1.3	41% (308/317)	(2011) Biochem. J. 436: 321
Serine protease from *Pyrococcus horikoshii*	4HXF	23.6	2.6	19% (236/613)	(2013) J. Biol. Chem. 288: 17884-17894
