# Supplementary material for: Crystal structure and functional characterization of a cold-active acetyl xylan esterase (PbAcE) from psychrophilic soil microbe Paenibacillus sp
Source: PLoS One. 2018 Oct 31;13(10):e0206260. doi: 10.1371/journal.pone.0206260 (PMC6209228; doi:10.1371/journal.pone.0206260)
Supplement: S1 File — (PDF) [file pone.0206260.s006.pdf]

Preliminary Full wwPDB X-ray Structure Validation Report (i)

Aug 13, 2018 – 06:56 PM JST

Deposition ID	:	D_1300008721
PDB ID	:	*(not yet assigned)*

This is a Preliminary Full wwPDB X-ray Structure Validation Report.

This report is produced by the wwPDB Deposition System during initial deposition but before annotation of the structure.

We welcome your comments at *validation@mail.wwpdb.org*
A user guide is available at
https://www.wwpdb.org/validation/2017/XrayValidationReportHelp
with specific help available everywhere you see the (i) symbol.

PRELIMINARY VALIDATION REPORT

The following versions of software and data (see references (i)) were used in the production of this report:

MolProbity	:	4.02b-467
Xtriage (Phenix)	:	1.13
EDS	:	rb-20031172
Percentile statistics	:	20171227.v01 (using entries in the PDB archive December 27th 2017)
Refmac	:	5.8.0158
CCP4	:	7.0 (Gargrove)
Ideal geometry (proteins)	:	Engh & Huber (2001)
Ideal geometry (DNA, RNA)	:	Parkinson et al. (1996)
Validation Pipeline (wwPDB-VP)	:	rb-20031172

1 Overall quality at a glance (i)

The following experimental techniques were used to determine the structure:
X-RAY DIFFRACTION

The reported resolution of this entry is 2.10 Å.

Percentile scores (ranging between 0-100) for global validation metrics of the entry are shown in the following graphic. The table shows the number of entries on which the scores are based.

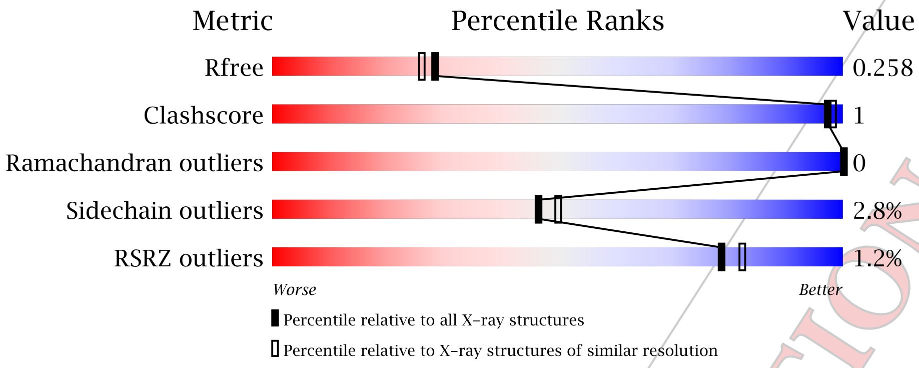

Metric	Whole archive (#Entries)	Similar resolution (#Entries, resolution range(Å))
R_{free}	111664	4608 (2.10-2.10)
Clashscore	122126	5109 (2.10-2.10)
Ramachandran outliers	120053	5059 (2.10-2.10)
Sidechain outliers	120020	5060 (2.10-2.10)
RSRZ outliers	108989	4497 (2.10-2.10)

The table below summarises the geometric issues observed across the polymeric chains and their fit to the electron density. The red, orange, yellow and green segments on the lower bar indicate the fraction of residues that contain outliers for >=3, 2, 1 and 0 types of geometric quality criteria. A grey segment represents the fraction of residues that are not modelled. The numeric value for each fraction is indicated below the corresponding segment, with a dot representing fractions <=5% The upper red bar (where present) indicates the fraction of residues that have poor fit to the electron density. The numeric value is given above the bar.

Mol	Chain	Length	Quality of chain
1	A	321	94% • •
1	B	321	.% 93% 6% •
1	C	321	.% 93% 5% •
1	D	321	.% 92% 7% •
1	E	321	2% 94% • •

Continued on next page...

PRELIMINARY VALIDATION REPORT

Continued from previous page...

Mol	Chain	Length	Quality of chain
1	F	321	2% 93% 5% •

PRELIMINARY VALIDATION REPORT

2 Entry composition (i)

There are 3 unique types of molecules in this entry. The entry contains 15833 atoms, of which 0 are hydrogens and 0 are deuteriums.

In the tables below, the ZeroOcc column contains the number of atoms modelled with zero occupancy, the AltConf column contains the number of residues with at least one atom in alternate conformation and the Trace column contains the number of residues modelled with at most 2 atoms.

- Molecule 1 is a protein called acetyl xylan esterase.

Mol	Chain	Residues	Atoms					ZeroOcc	AltConf	Trace
1	A	316	Total 2510	C 1607	N 428	O 461	S 14	0	0	0
1	B	316	Total 2510	C 1607	N 428	O 461	S 14	0	0	0
1	C	316	Total 2510	C 1607	N 428	O 461	S 14	0	0	0
1	D	316	Total 2510	C 1607	N 428	O 461	S 14	0	0	0
1	E	316	Total 2510	C 1607	N 428	O 461	S 14	0	0	0
1	F	316	Total 2510	C 1607	N 428	O 461	S 14	0	0	0

- Molecule 2 is ZINC ION (three-letter code: ZN) (formula: Zn).

Mol	Chain	Residues	Atoms		ZeroOcc	AltConf
2	G	1	Total 1	Zn 1	0	0
2	G	1	Total 1	Zn 1	0	0
2	G	1	Total 1	Zn 1	0	0
2	G	1	Total 1	Zn 1	0	0
2	G	1	Total 1	Zn 1	0	0
2	G	1	Total 1	Zn 1	0	0

- Molecule 3 is water.

PRELIMINARY VALIDATION REPORT

Mol	Chain	Residues	Atoms		ZeroOcc	AltConf
			Total	O		
3	S	767	767	767	0	0

PRELIMINARY VALIDATION REPORT

3 Residue-property plots (i)

These plots are drawn for all protein, RNA and DNA chains in the entry. The first graphic for a chain summarises the proportions of the various outlier classes displayed in the second graphic. The second graphic shows the sequence view annotated by issues in geometryand electron density. Residues are color-coded according to the number of geometric quality criteria for which they contain at least one outlier: green = 0, yellow = 1, orange = 2 and red = 3 or more. A red dot above a residue indicates a poor fit to the electron density (RSRZ > 2). Stretches of 2 or more consecutive residues without any outlier are shown as a green connector. Residues present in the sample, but not in the model, are shown in grey.

- Molecule 1: acetyl xylan esterase

Chain A:

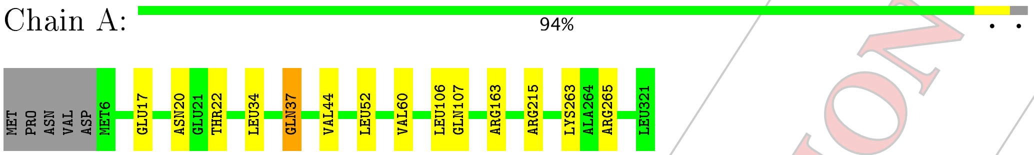

- Molecule 1: acetyl xylan esterase

Chain B:

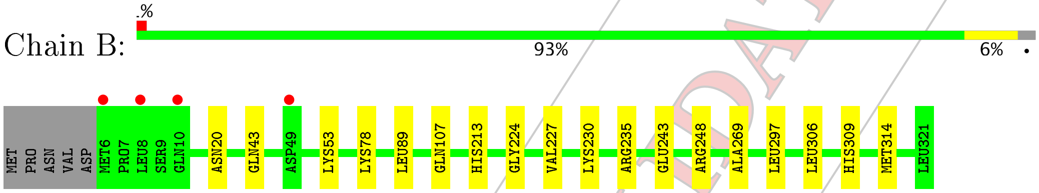

- Molecule 1: acetyl xylan esterase

Chain C:

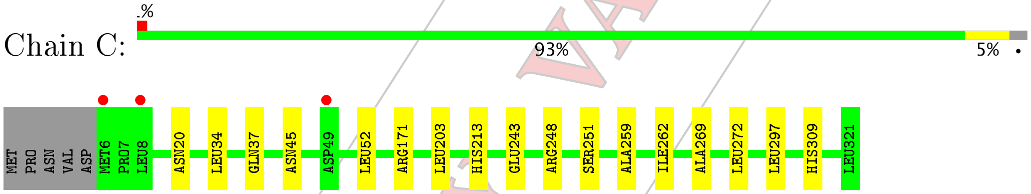

- Molecule 1: acetyl xylan esterase

Chain D:

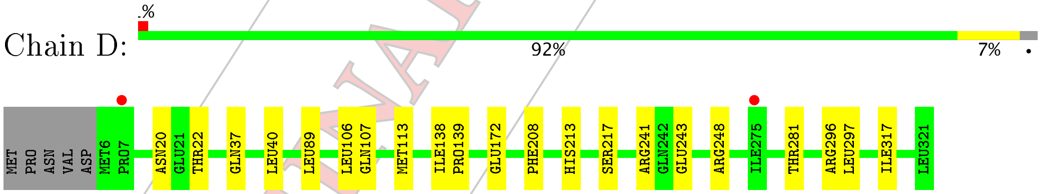

- Molecule 1: acetyl xylan esterase

Chain E:

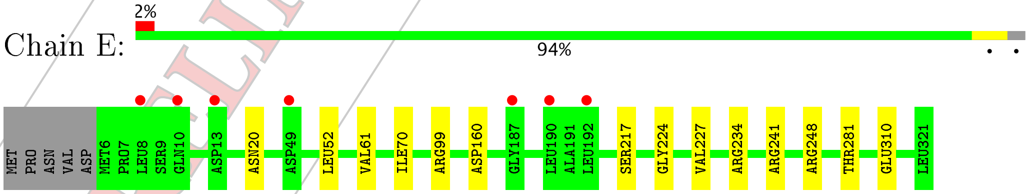

PRELIMINARY VALIDATION REPORT

• Molecule 1: acetyl xylan esterase

Chain F:

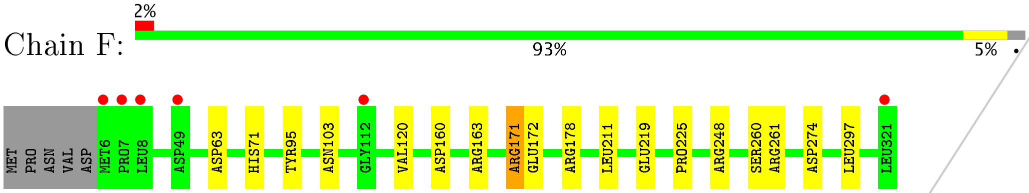

PRELIMINARY VALIDATION REPORT

4 Data and refinement statistics (i)

Property	Value	Source
Space group	C 1 2 1	Depositor
Cell constants<br>a, b, c, α, β, γ	153.80Å 141.66Å 105.37Å<br>90.00° 104.49° 90.00°	Depositor
Resolution (Å)	49.39 – 2.10<br>35.09 – 2.10	Depositor<br>EDS
% Data completeness<br>(in resolution range)	96.1 (49.39-2.10)<br>96.1 (35.09-2.10)	Depositor<br>EDS
R_{merge}	(Not available)	Depositor
R_{sym}	(Not available)	Depositor
$< I/\sigma(I) >$ [1]	11.15 (at 2.10Å)	Xtriage
Refinement program	REFMAC 5.8.0189	Depositor
R, R_{free}	0.198 , 0.254<br>0.206 , 0.258	Depositor<br>DCC
R_{free} test set	6247 reflections (5.13%)	wwPDB-VP
Wilson B-factor (Å^2)	28.0	Xtriage
Anisotropy	0.189	Xtriage
Bulk solvent k_{sol}(e/Å^3), B_{sol}(Å^2)	0.37 , 40.6	EDS
L-test for twinning[2]	$< \|L\| > = 0.47$, $< L^2 > = 0.30$	Xtriage
Estimated twinning fraction	No twinning to report.	Xtriage
F_o,F_c correlation	0.94	EDS
Total number of atoms	15833	wwPDB-VP
Average B, all atoms (Å^2)	36.0	wwPDB-VP

Xtriage's analysis on translational NCS is as follows: *The largest off-origin peak in the Patterson function is 3.35% of the height of the origin peak. No significant pseudotranslation is detected.*

[1] Intensities estimated from amplitudes.

[2] Theoretical values of $< |L| >$, $< L^2 >$ for acentric reflections are 0.5, 0.333 respectively for untwinned datasets, and 0.375, 0.2 for perfectly twinned datasets.

PRELIMINARY VALIDATION REPORT

5 Model quality (i)

5.1 Standard geometry (i)

Bond lengths and bond angles in the following residue types are not validated in this section: ZN

The Z score for a bond length (or angle) is the number of standard deviations the observed value is removed from the expected value. A bond length (or angle) with $|Z| > 5$ is considered an outlier worth inspection. RMSZ is the root-mean-square of all Z scores of the bond lengths (or angles).

Mol	Chain	Bond lengths		Bond angles	
		RMSZ	#|Z| >5	RMSZ	#|Z| >5
1	A	0.89	0/2585	0.92	5/3516 (0.1%)
1	B	0.85	0/2585	0.89	4/3516 (0.1%)
1	C	0.90	1/2585 (0.0%)	0.91	1/3516 (0.0%)
1	D	0.91	0/2585	0.92	2/3516 (0.1%)
1	E	0.87	0/2585	0.92	6/3516 (0.2%)
1	F	0.82	0/2585	0.92	5/3516 (0.1%)
All	All	0.87	1/15510 (0.0%)	0.91	23/21096 (0.1%)

All (1) bond length outliers are listed below:

Mol	Chain	Res	Type	Atoms	Z	Observed(Å)	Ideal(Å)
1	C	251	SER	CB-OG	5.11	1.48	1.42

All (23) bond angle outliers are listed below:

Mol	Chain	Res	Type	Atoms	Z	Observed(°)	Ideal(°)
1	E	160	ASP	CB-CG-OD1	9.05	126.45	118.30
1	E	160	ASP	CB-CG-OD2	-7.61	111.45	118.30
1	F	171	ARG	NE-CZ-NH1	7.41	124.00	120.30
1	B	235	ARG	NE-CZ-NH2	6.38	123.49	120.30
1	F	160	ASP	CB-CG-OD1	6.27	123.94	118.30
1	D	248	ARG	NE-CZ-NH1	6.13	123.36	120.30
1	F	274	ASP	CB-CG-OD1	5.90	123.61	118.30
1	F	178	ARG	NE-CZ-NH1	5.84	123.22	120.30
1	E	99	ARG	NE-CZ-NH1	5.84	123.22	120.30
1	B	235	ARG	NE-CZ-NH1	-5.70	117.45	120.30
1	C	248	ARG	NE-CZ-NH1	5.67	123.14	120.30
1	B	248	ARG	NE-CZ-NH1	5.52	123.06	120.30
1	E	234	ARG	NE-CZ-NH2	-5.52	117.54	120.30
1	A	265	ARG	NE-CZ-NH1	5.46	123.03	120.30

Continued on next page...

PRELIMINARY VALIDATION REPORT

Continued from previous page...

Mol	Chain	Res	Type	Atoms	Z	Observed(^{o})	Ideal(^{o})
1	F	248	ARG	NE-CZ-NH2	5.42	123.01	120.30
1	E	248	ARG	NE-CZ-NH1	5.32	122.96	120.30
1	B	248	ARG	NE-CZ-NH2	-5.29	117.66	120.30
1	D	296	ARG	NE-CZ-NH1	5.27	122.94	120.30
1	A	163	ARG	NE-CZ-NH2	-5.25	117.67	120.30
1	A	215	ARG	NE-CZ-NH2	-5.11	117.74	120.30
1	A	265	ARG	NE-CZ-NH2	-5.08	117.76	120.30
1	E	241	ARG	NE-CZ-NH2	-5.06	117.77	120.30
1	A	215	ARG	NE-CZ-NH1	5.06	122.83	120.30

There are no chirality outliers.

There are no planarity outliers.

5.2 Too-close contacts (i)

In the following table, the Non-H and H(model) columns list the number of non-hydrogen atoms and hydrogen atoms in the chain respectively. The H(added) column lists the number of hydrogen atoms added and optimized by MolProbity. The Clashes column lists the number of clashes within the asymmetric unit, whereas Symm-Clashes lists symmetry related clashes.

Mol	Chain	Non-H	H(model)	H(added)	Clashes	Symm-Clashes
1	A	2510	0	2415	4	0
1	B	2510	0	2415	5	0
1	C	2510	0	2415	3	0
1	D	2510	0	2415	6	0
1	E	2510	0	2415	4	0
1	F	2510	0	2415	6	0
2	G	6	0	0	0	0
3	S	767	0	0	5	0
All	All	15833	0	14490	26	0

The all-atom clashscore is defined as the number of clashes found per 1000 atoms (including hydrogen atoms). The all-atom clashscore for this structure is 1.

All (26) close contacts within the same asymmetric unit are listed below, sorted by their clash magnitude.

Atom-1	Atom-2	Interatomic distance (Å)	Clash overlap (Å)
1:A:37:GLN:HA	1:A:37:GLN:HE21	1.67	0.58
1:C:259:ALA:HA	1:C:262:ILE:HD12	1.88	0.56
1:E:224:GLY:O	1:E:227:VAL:HG12	2.05	0.55

Continued on next page...

PRELIMINARY VALIDATION REPORT

Continued from previous page...

Atom-1	Atom-2	Interatomic distance (Å)	Clash overlap (Å)
1:A:44:VAL:HG22	1:A:60:VAL:HG22	1.90	0.54
1:A:37:GLN:HE21	1:A:37:GLN:CA	2.22	0.53
1:E:310:GLU:CD	1:F:103:ASN:HD22	2.15	0.50
1:B:224:GLY:O	1:B:227:VAL:HG22	2.15	0.47
1:C:269:ALA:HB2	1:C:309:HIS:CE1	2.50	0.46
1:D:89:LEU:HD21	1:D:317:ILE:HD13	1.97	0.46
1:F:71:HIS:HD2	3:S:706:HOH:O	1.99	0.46
1:B:53:LYS:O	1:B:78:LYS:HE3	2.16	0.46
1:B:107:GLN:HB2	1:B:314:MET:CE	2.46	0.45
1:D:113:MET:HE1	1:D:317:ILE:HG21	1.99	0.44
1:F:95:TYR:CD2	1:F:225:PRO:HG3	2.52	0.44
1:A:106:LEU:HD21	1:D:107:GLN:HA	2.00	0.44
1:C:171:ARG:NH1	3:S:343:HOH:O	2.39	0.44
1:F:261:ARG:CZ	3:S:712:HOH:O	2.66	0.44
1:B:230:LYS:NZ	3:S:528:HOH:O	2.48	0.44
1:E:61:VAL:HA	1:E:70:ILE:O	2.18	0.44
1:F:63:ASP:O	1:F:163:ARG:NH2	2.40	0.43
1:B:269:ALA:HB2	1:B:309:HIS:CE1	2.53	0.43
1:D:89:LEU:HD21	1:D:317:ILE:CD1	2.49	0.42
1:D:138:ILE:HG23	1:D:139:PRO:HD2	2.02	0.42
1:D:217:SER:OG	1:D:281:THR:HG21	2.20	0.42
1:E:217:SER:OG	1:E:281:THR:HG21	2.20	0.42
1:F:120:VAL:HG22	3:S:362:HOH:O	2.22	0.40

There are no symmetry-related clashes.

5.3 Torsion angles (i)

5.3.1 Protein backbone (i)

In the following table, the Percentiles column shows the percent Ramachandran outliers of the chain as a percentile score with respect to all X-ray entries followed by that with respect to entries of similar resolution.

The Analysed column shows the number of residues for which the backbone conformation was analysed, and the total number of residues.

Mol	Chain	Analysed	Favoured	Allowed	Outliers	Percentiles	
1	A	314/321 (98%)	303 (96%)	11 (4%)	0	100	100
1	B	314/321 (98%)	305 (97%)	9 (3%)	0	100	100

Continued on next page...

PRELIMINARY VALIDATION REPORT

Continued from previous page...

Mol	Chain	Analysed	Favoured	Allowed	Outliers	Percentiles	
1	C	314/321 (98%)	299 (95%)	15 (5%)	0	100	100
1	D	314/321 (98%)	306 (98%)	8 (2%)	0	100	100
1	E	314/321 (98%)	301 (96%)	13 (4%)	0	100	100
1	F	314/321 (98%)	299 (95%)	15 (5%)	0	100	100
All	All	1884/1926 (98%)	1813 (96%)	71 (4%)	0	100	100

There are no Ramachandran outliers to report.

5.3.2 Protein sidechains (i)

In the following table, the Percentiles column shows the percent sidechain outliers of the chain as a percentile score with respect to all X-ray entries followed by that with respect to entries of similar resolution.

The Analysed column shows the number of residues for which the sidechain conformation was analysed, and the total number of residues.

Mol	Chain	Analysed	Rotameric	Outliers	Percentiles	
1	A	262/267 (98%)	254 (97%)	8 (3%)	43	45
1	B	262/267 (98%)	255 (97%)	7 (3%)	48	51
1	C	262/267 (98%)	252 (96%)	10 (4%)	36	36
1	D	262/267 (98%)	251 (96%)	11 (4%)	32	32
1	E	262/267 (98%)	260 (99%)	2 (1%)	83	88
1	F	262/267 (98%)	256 (98%)	6 (2%)	53	58
All	All	1572/1602 (98%)	1528 (97%)	44 (3%)	47	50

All (44) residues with a non-rotameric sidechain are listed below:

Mol	Chain	Res	Type
1	A	17	GLU
1	A	20	ASN
1	A	22	THR
1	A	34	LEU
1	A	37	GLN
1	A	52	LEU
1	A	107	GLN
1	A	263	LYS
1	B	20	ASN

Continued on next page...

PRELIMINARY VALIDATION REPORT

Continued from previous page...

Mol	Chain	Res	Type
1	B	43	GLN
1	B	89	LEU
1	B	213	HIS
1	B	243	GLU
1	B	297	LEU
1	B	306	LEU
1	C	20	ASN
1	C	34	LEU
1	C	37	GLN
1	C	45	ASN
1	C	52	LEU
1	C	203	LEU
1	C	213	HIS
1	C	243	GLU
1	C	272	LEU
1	C	297	LEU
1	D	20	ASN
1	D	22	THR
1	D	37	GLN
1	D	40	LEU
1	D	106	LEU
1	D	172	GLU
1	D	208	PHE
1	D	213	HIS
1	D	241	ARG
1	D	243	GLU
1	D	297	LEU
1	E	20	ASN
1	E	52	LEU
1	F	171	ARG
1	F	172	GLU
1	F	211	LEU
1	F	219	GLU
1	F	260	SER
1	F	297	LEU

Some sidechains can be flipped to improve hydrogen bonding and reduce clashes. All (4) such sidechains are listed below:

Mol	Chain	Res	Type
1	A	37	GLN
1	C	12	GLN
1	E	12	GLN

Continued on next page...

PRELIMINARY VALIDATION REPORT

Continued from previous page...

Mol	Chain	Res	Type
1	F	37	GLN

5.3.3 RNA (i)

There are no RNA molecules in this entry.

5.4 Non-standard residues in protein, DNA, RNA chains (i)

There are no non-standard protein/DNA/RNA residues in this entry.

5.5 Carbohydrates (i)

There are no carbohydrates in this entry.

5.6 Ligand geometry (i)

Of 6 ligands modelled in this entry, 6 are monoatomic - leaving 0 for Mogul analysis.

There are no bond length outliers.

There are no bond angle outliers.

There are no chirality outliers.

There are no torsion outliers.

There are no ring outliers.

No monomer is involved in short contacts.

5.7 Other polymers (i)

There are no such residues in this entry.

5.8 Polymer linkage issues (i)

There are no chain breaks in this entry.

PRELIMINARY VALIDATION REPORT

6 Fit of model and data (i)

6.1 Protein, DNA and RNA chains (i)

In the following table, the column labelled '#RSRZ> 2' contains the number (and percentage) of RSRZ outliers, followed by percent RSRZ outliers for the chain as percentile scores relative to all X-ray entries and entries of similar resolution. The OWAB column contains the minimum, median, 95th percentile and maximum values of the occupancy-weighted average B-factor per residue. The column labelled 'Q< 0.9' lists the number of (and percentage) of residues with an average occupancy less than 0.9.

Mol	Chain	Analysed	<RSRZ>	#RSRZ>2			OWAB(Å^2)	Q<0.9
1	A	316/321 (98%)	-0.32	0	100	100	21, 32, 52, 65	0
1	B	316/321 (98%)	-0.15	4 (1%)	77	80	22, 34, 53, 83	0
1	C	316/321 (98%)	-0.27	3 (0%)	84	86	22, 31, 51, 84	0
1	D	316/321 (98%)	-0.22	2 (0%)	89	91	22, 33, 52, 73	0
1	E	316/321 (98%)	-0.12	7 (2%)	62	67	21, 33, 54, 76	0
1	F	316/321 (98%)	0.01	6 (1%)	66	71	23, 42, 62, 90	0
All	All	1896/1926 (98%)	-0.18	22 (1%)	79	82	21, 34, 55, 90	0

All (22) RSRZ outliers are listed below:

Mol	Chain	Res	Type	RSRZ
1	F	8	LEU	4.4
1	F	6	MET	4.0
1	B	6	MET	3.9
1	E	49	ASP	3.7
1	C	6	MET	3.7
1	F	49	ASP	2.9
1	E	13	ASP	2.9
1	C	49	ASP	2.7
1	D	275	ILE	2.5
1	E	190	LEU	2.4
1	D	7	PRO	2.4
1	E	187	GLY	2.3
1	C	8	LEU	2.2
1	B	8	LEU	2.2
1	F	7	PRO	2.1
1	E	192	LEU	2.1
1	B	49	ASP	2.1
1	E	10	GLN	2.1
1	F	112	GLY	2.1

Continued on next page...

PRELIMINARY VALIDATION REPORT

Continued from previous page...

Mol	Chain	Res	Type	RSRZ
1	B	10	GLN	2.0
1	E	8	LEU	2.0
1	F	321	LEU	2.0

6.2 Non-standard residues in protein, DNA, RNA chains ⓘ

There are no non-standard protein/DNA/RNA residues in this entry.

6.3 Carbohydrates ⓘ

There are no carbohydrates in this entry.

6.4 Ligands ⓘ

In the following table, the Atoms column lists the number of modelled atoms in the group and the number defined in the chemical component dictionary. The B-factors column lists the minimum, median, 95th percentile and maximum values of B factors of atoms in the group. The column labelled '$Q< 0.9$' lists the number of atoms with occupancy less than 0.9.

Mol	Type	Chain	Res	Atoms	RSCC	RSR	B-factors($Å^2$)	Q<0.9
2	ZN	G	2	1/?	0.94	0.07	67,67,67,67	0
2	ZN	G	6	1/?	0.95	0.07	71,71,71,71	0
2	ZN	G	3	1/?	0.95	0.04	70,70,70,70	0
2	ZN	G	5	1/?	0.96	0.07	67,67,67,67	0
2	ZN	G	4	1/?	0.97	0.06	71,71,71,71	0
2	ZN	G	1	1/?	0.98	0.12	63,63,63,63	0

6.5 Other polymers ⓘ

There are no such residues in this entry.

PRELIMINARY VALIDATION REPORT
